# Novel polymorphic *AluYb8* insertion in the *WNK1* gene is associated with blood pressure variation in Europeans

**DOI:** 10.1002/humu.21508

**Published:** 2011-04-21

**Authors:** Margus Putku, Katrin Kepp, Elin Org, Siim Sõber, David Comas, Margus Viigimaa, Gudrun Veldre, Peeter Juhanson, Pille Hallast, Neeme Tõnisson, Sue Shaw-Hawkins, Mark J Caulfield, Elza Khusnutdinova, Viktor Kožich, Patricia B Munroe, Maris Laan, HYPEST, BRIGHT

**Affiliations:** 1 Human Molecular Genetics Research Group, Institute of Molecular and Cell Biology, University of TartuTartu, Estonia; 2 Institute of Evolutionary Biology (UPF-CSIC), CEXS-UPF-PRBB, Universitat Pompeu FabraBarcelona, Spain; 3 Centre of Cardiology, North Estonia Medical CentreTallinn, Estonia; 4 Tallinn University of Technology, Department of Biomedical Engineering, Chair of Medical PhysicsTallinn, Estonia; 5 Department of Cardiology, University of TartuTartu, Estonia; 6 Clinical Pharmacology and The Genome Centre, William Harvey Research Institute, Barts and The London School of Medicine and Dentistry, Queen Mary University of LondonLondon EC1M 6BQ, United Kingdom; 7 Institute of Biochemistry and Genetics, Ufa Science Center, Russian Academy of SciencesUfa, Bashkortostan, Russia; 8 Institute of Inherited Metabolic Diseases, Charles University—First Faculty of MedicinePrague, Czech Republic

**Keywords:** *WNK1*, polymorphism screening, *AluYb8*, blood pressure, meta-analysis

## Abstract

Mutations in *WNK1* and *WNK4* cause familial hypertension, the Gordon syndrome. *WNK1* and *WNK4* conserved noncoding regions were targeted to polymorphism screening using DHPLC and DGGE. The scan identified an undescribed polymorphic *AluYb8* insertion in *WNK1* intron 10. Screening in primates revealed that this *Alu*-insertion has probably occurred in human lineage. Genotyping in 18 populations from Europe, Asia, and Africa (*n* = 854) indicated an expansion of the *WNK1 AluYb8* bearing chromosomes out of Africa. The allele frequency in Sub-Saharan Africa was ∼3.3 times lower than in other populations (4.8 vs. 15.8%; *P* = 9.7 × 10^−9^). Meta-analysis across three European sample sets (*n* = 3,494; HYPEST, Estonians; BRIGHT, the British; CADCZ, Czech) detected significant association of the *WNK1 AluYb8* insertion with blood pressure (BP; systolic BP, *P* = 4.03 × 10^−3^, effect 1.12; diastolic BP, *P* = 1.21 × 10^−2^, effect 0.67). Gender-stratified analysis revealed that this effect might be female-specific (*n* = 2,088; SBP, *P* = 1.99 × 10^−3^, effect 1.59; DBP *P* = 3.64 × 10^−4^, effect 1.23; resistant to Bonferroni correction), whereas no statistical support was identified for the association with male BP (*n* = 1,406). In leucocytes, the expressional proportions of the full-length *WNK1* transcript and the splice-form skipping exon 11 were significantly shifted in *AluYb8* carriers compared to noncarriers. The *WNK1 AluYb8* insertion might affect human BP via altering the profile of alternatively spliced transcripts. Hum Mutat 32:1–9, 2011. © 2011 Wiley-Liss, Inc.

## Introduction

Essential hypertension is a complex disease promoted by an unfavorable combination of person's life style and heritable factors. It is a significant health risk leading to other cardiovascular and renal diseases. Genetic studies of monogenic, Mendelian forms of hypo- and hypertension have identified ∼20 rare mutations in blood pressure regulating genes with a strong effect on the phenotype [Lifton et al., 2001; Vehaskari, 2007]. Although these rare mutations do not explain blood pressure variation in the general population, the identified genes are promising targets for functional, physiological and genetic studies of essential hypertension [Ji et al., 2008].

Serine/threonine protein kinase family members WNK1 (MIM♯ 605232) and WNK4 (MIM♯ 601844) [Verissimo and Jordan, 2001; Xu et al., 2000] are involved in the development of a Mendelian form of hypertension, pseudohypoaldosteronism type II, or the Gordon syndrome [Wilson et al., 2001, 2003]. The syndrome is caused either by large deletions (two identified variants: 22 and 42 kb) in the first intron of *WNK1* or by nonsynonymous substitutions in *WNK4* (four described mutations). Although *WNK1* and *WNK4* are expressed in multiple tissues, their major role is to regulate the transport of sodium and potassium ions in distal convoluted tubule and cortical collecting duct of nephrons, and thereby to contribute to blood pressure determination [Verissimo and Jordan, 2001; Wilson et al., 2001]. The human *WNK4* gene (19 exons) spans ∼16 kb on chromosome 17q21.31. The human *WNK1* gene (29 exons) covers ∼160 kb on chromosome 12p13 and codes for multiple transcripts initiated by alternative promoters [Delaloy et al., 2003; Wilson et al., 2001; Xu et al., 2000]. Two major WNK1 isoforms have been described: a long isoform (L-WNK1) with complete kinase domain and a short kidney-specific isoform (KS-WNK1), which is kinase-deficient [Xu et al., 2000]. Although multiple alternative splice-forms of *WNK1* have been identified, the function of individual transcripts is yet to be determined. In addition to the identification of rare variants in *WNK1* and *WNK4* responsible for the Gordon syndrome, common single nucleotide polymorphisms (SNPs) in these genes have been associated with blood pressure variation and susceptibility to hypertension in general population among adults as well as children [Kokubo et al., 2004; Newhouse et al., 2005, 2009; Osada et al., 2009; Tobin et al., 2005, 2008]. SNPs in *WNK1* also affect the response of thiazide diuretics treatment on patient's blood pressure [Turner et al., 2005].

Although monogenic diseases are usually caused by rare variants located in the coding sequence of a gene, common diseases are rather considered to result from genetic variation in gene regulatory elements altering the expressional profile of the locus [Pastinen and Hudson, 2004; Visel et al., 2009]. As gene regulatory elements tend to map within evolutionarily conserved segments of the genome [Elgar and Vavouri, 2008; Hardison, 2000], these regions have a potential to harbor polymorphisms contributing to the susceptibility to common traits including essential hypertension.

The aim of the current study was to screen the evolutionarily conserved noncoding regions of *WNK1* and *WNK4* to identify novel polymorphisms potentially affecting blood pressure in general population. Variant screening resulted in the identification of a novel human-specific polymorphic *AluYb8* insertion in *WNK1* intron 10. This *Alu*-insertion was targeted to further evolutionary and population genetic analysis, as well as was also explored for association with blood pressure and its effect on the transcriptional profile of the *WNK1* gene in leucocytes.

## Materials and Methods

### In Silico Analysis of Conserved Noncoding Regions in *WNK1* and *WNK4*

Conserved noncoding regions (CNRs) in *WNK1* and *WNK4* were screened using the Web-based VISTA software (http://genome.lbl.gov/vista/index.shtml) with the proposed default parameters (cutoff criteria: 100-bp sliding window; sequence identity ≥70%; comparison with rat and mouse). The analyzed loci spanned from 10 kb upstream to 10 kb downstream of *WNK1* (12p13.3; coordinates 722,486–900,879, NCBI Build 36.1, hg18) and *WNK4* (17q21.31; coordinates 38,176,222–38,212,587, NCBI Build 36.1, hg18). All VISTA regions that had any overlap with annotated genes track at UCSC Genome Browser (http://genome.ucsc.edu/) were excluded as potential coding regions. Polymorphism discovery was targeted to CNRs with sequence identity >70% between human and rodents, length of the region 50–300 bp, and location >200 bp from the nearest exon (Supp. Table S1).

### Screening for Novel Polymorphisms in *WNK1* and *WNK4* Conserved Noncoding Regions

In total, 40 CNRs (*n* = 29 in *WNK1*; *n* = 11 in *WNK4*) were selected for polymorphism screening, which was conducted either by *Denaturing Gradient Gel Electrophoresis* (DGGE; INGENYphorU-2 × 2 system, Ingeny International BV, Goes, The Netherlands) and/or *Denaturing High-Performance Liquid Chromatography* method (DHPLC; Wave Technologies Inc., Herndon, VA). In the design of the DGGE and DHPLC assays and in establishing the experimental conditions, the manufacturers' recommendations were followed. Details of the assays are given in Supp. Text S1. The design of both DGGE and DHPLC assays was unsuccessful for seven CNRs in *WNK1* and two CNRs in *WNK4* due to failure in primer design (inappropriate primer *T*_*m*_ or more than two *T*_*m*_ melting points for the region of interest) or a negative result in the genome test (Supp. Table S1). The genome test was applied to confirm the unique binding of a tested primer in the genome. The rest of the 31 selected CNRs were screened for polymorphisms either by DHPLC (seven regions in *WNK1*; five in *WNK4*), by DGGE (seven in *WNK1*; one in *WNK4*) or by both assays (eight in *WNK1*; three in *WNK4*; Supp. Table S1). Primers for DHPLC and DGGE assays are given in Supp. Tables S2 and S3, respectively.

The average length of the CNR segments selected for polymorphism screening was 145 bp (range: 68–291 bp) and PCR fragments was 360 bp (range: 245–487 bp). Genomic DNAs of essential hypertension patients from two Eastern European studies (*n* = 22 from HYPEST; *n* = 24 from CADCZ; detailed description below) were targeted to polymorphism screening by DGGE (individual DNAs) and/or DHPLC (pools of DNA from three patients). PCR products exhibiting evidence for the presence of a polymorphism were sequenced on both forward and reverse orientations. Polymorphisms were identified using BioEdit Sequence Alignment Editor (T. Hall, Department of Microbiology, North Carolina State University).

### Genotyping of the *WNK1 AluYb8* in General Human Population Samples

**Figure 1:**
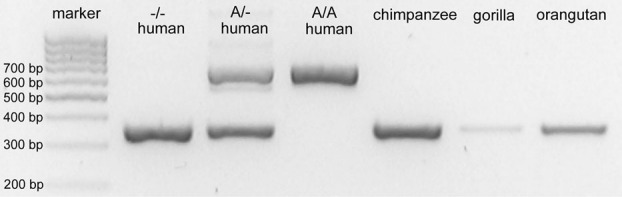
Detection of the presence of *WNK1* intron 10 *AluYb8* insertion in primates. Agarose gel (3%) electrophoresis of *WNK1* intron 10 PCR products amplified from human, chimpanzee, gorilla, and orangutan genomic DNAs. In humans, alternative genotype carriers are shown: wild-type homozygote without *AluYb8* insertion (−/−, PCR product 353 bp); heterozygous (A/−) and homozygous (A/A, PCR product 660 bp) carriers of the insertion

For large-scale genotyping of *WNK1 AluYb8* in humans PCR followed by standard agarose gel electrophoresis was used. The primer design (WNK1_Alu_F: 5′-GGGTAACCAACCCTTGAAGTAGG-3′; WNK1_Alu_R: 5′-GGGTACTTCTCAAGTGATTAGGAGGA-3′) was carried out using the Web-based program Primer3 [Rozen and Skaletsky, 2000]. Quality control of the genotyping by agarose gel electrophoresis was assured by including previously resequenced positive controls representing alternative genotype carriers on each gel: wild-type (PCR product 353 bp); heterozygous (PCR products 353 and 660 bp) and homozygous (PCR product 660 bp) individuals for the *AluYb8* insertion (Fig. 1; Supp. Figs. S1 and S2). The distribution of the *WNK1 AluYb8* insertion was studied in six European (Estonians, *n* = 100; Czech, *n* = 50; CEPH, *n* = 30; the Basque, *n* = 50; Catalans, *n* = 41; Spanish Gypsies, *n* = 50), four Asian (Koreans, *n* = 43; Chinese Han, *n* = 25; Tatars, *n* = 47; Bashkir, *n* = 47), and eight African populations (Tunisians, *n* = 48; Algerians, *n* = 48; Moroccans, *n* = 84; Mandenkalu, *n* = 24; Saharawi, *n* = 50; Gabon Bantus, *n* = 50; Gabon Pygmies, *n* = 50; Tanzanians, *n* = 17).

### Conservation of the *WNK1 AluYb8* Insertion in Primates

The presence of the *WNK1 AluYb8* insertion was ascertained for a gorilla (*Gorilla gorilla;* primary cell line AG05251B, purchased from ECACC), for an orangutan (*Pongo pygmaeus*; primary cell line AG12256, purchased from ECACC) and for 11 western chimpanzees (*Pan troglodytes verus*) using identical PCR setup as in human genotyping. DNA sample of one chimpanzee originates from a wild-born male specimen (Pino) from Tallinn Zoo, Estonia. Ten samples of wild-caught and unrelated animals (Annaclara, Frits, Hilko, Louise, Marco, Oscar, Regina, Socrates, Sonja, and Yoran) are from the collection stored at the Max Planck Institute for Evolutionary Anthropology, Leipzig, Germany, and were kindly shared by Dr. Svante Pääbo. This sample collection is described in detail elsewhere [Becquet et al., 2007; Ptak et al., 2004].

Ancestral sequence of the targeted genomic region (*WNK1* exon10–intron10–exon11) was assessed by the comparative sequencing of the genomic DNA from *WNK1 AluYb8* insertion noncarrier (−/−) and carrier (Alu/Alu) human homozygotes as well as from a chimpanzee (Pino). Sequencing primers are listed in Supp. Table S4. PCR cycling conditions, product purification, and sequencing have been described elsewhere [Hallast et al., 2005]. Sequences were aligned using Web-based global alignment program ClustalW2 (http://www.ebi.ac.uk/Tools/clustalw2/). Human WNK1 alternative sequences of the region including exon 10/intron 10/exon 11 (without and with *AluYb8* insertion) were compared with available genome sequences from multiple species using the BLAST tool blastn (http://blast.ncbi.nlm.nih.gov/Blast.cgi). The searches were performed against the following sequence databases: NCBI Genomes, Whole-Genome-Shotgun Sequences. Nucleotide substitution rates between human and chimpanzee were calculated as the percentage of the number of substitutions divided with the total number of aligned nucleotides in the specific genomic region. The number of substitutions and the total number of aligned nucleotides were calculated using EMBOSS stretcher (http://emboss.sourceforge.net/) [Rice et al., 2000].

### Stage 1 Association Analysis in HYPEST

In Stage 1, the association of the *WNK1 AluYb8* insertion with BP was addressed using HYPEST (HYPertension in ESTonia) case–cohort sample collection (Table 1; recruitment details in Supp. Text S1). The HYPEST study has been approved by the Ethics Committee on Human Research of University of Tartu (no. 122/13, 22.12.2003; 137/20, 25.04.2005) and it was carried out in compliance with the Helsinki Declaration. All the participants have given their written informed consent. HYPEST subjects were recruited across Estonia during 2004–2007 (1,823 individuals; age range: 18–85 years) with the aim to analyze genetic–epidemiological risk factors for essential hypertension and related cardiovascular disease in Estonian population. In the current study, the total number of genotyped HYPEST subjects was *n* = 1,747. At the recruitment, the resting BP of each participant has been measured by trained clinicians using a standard mercury column sphygmomanometer and size-adjusted cuffs. HYPEST individuals possessed a documented history of multiple systolic and diastolic BP readings (on average, 4.31 readings per individual during mean 3.17 years). For the analysis, the median across the longitudinal BP readings as well as the median of the subject's age during the readings were used.

**Table 1:** Phenotypic Parameters of Study Subjects in the Analysis with Systolic (SBP) and Diastolic (DBP) Blood Pressure and Hypertension (HYP)

	HYPEST			CADCZ			BRIGHT	
			
	Population sample^a^	Essential hypertension^b^		Population sample^a^	Essential hypertension^b^		Family-based hypertension^c^	
					
Parameter (Mean ± SD)		Cases	Controls		Cases	Controls	Cases	Controls
No. of individuals	1,211	673	601	644	266	480	2,242	1,639
(male/female)	(408/803)	(228/445)	(162/439)	(361/283)	(180/86)	(229/251)	(922/1,320)	(637/1,002)
Age at recruitment (y)	44.8 ± 12.5	56.0 ± 9.5	38.9 ± 9.0	47.8 ± 11.0	55.5 ± 6.5	45.9 ± 11.1	57.2 ± 10.8	58.8 ± 9.0
Age at onset of disease (years)	na	44.0 ± 12.7	na	na	46.5 ± 9.6	na	46.8 ± 10.3	na
BMI (kg/m^2^)	26.7 ± 4.8	30.3 ± 5.1	24.6 ± 4.3	26.1 ± 4.0	29.3 ± 4.5	25.6 ± 3.9	27.4 ± 3.9	25.3 ± 3.2
SBP (mmHg)	141.0 ± 19.0	144.2 ± 17.6	127.8 ± 8.0	126.0 ± 14.4	146.8 ± 17.7	122.0 ± 10.2	154.2 ± 20.8	123.1 ± 10.5
DBP (mmHg)	87.2 ± 11.0	88.3 ± 10.6	80.8 ± 6.4	80.4 ± 9.4	90.6 ± 9.3	78.2 ± 8.0	94.1 ± 11.1	76.5 ± 7.1
Antihypertensive treatment (% of subjects)	0.0%	78.5%	0.0%	0.0%	85.0%	0.0%	100%	0.0%

a Subjects from Estonian (HYPEST) and Czech (CADCZ) populations not receiving antihypertensive medication and used in association analysis with SBP and DBP.

b Cases: subjects under antihypertensive treatment or untreated subjects SBP ≥160 mmHg and/or DBP ≥100 mmHg; Controls: subjects with SBP ≤140 mmHg and DBP ≤90 mmHg, receiving no antihypertensive medication.

c Cases: patients from severely hypertensive families, under antihypertensive treatment and with BP ≥150/100 mmHg based on one reading or ≥145/95 mmHg based on the mean of three readings; Controls: subjects with BP <140/90 mmHg, receiving no antihypertensive medication. SD, standard deviation; BMI, body mass index (kg/m^2^); SBP, DBP, systolic and diastolic blood pressure; y, age in years; na, not applicable.

Association analysis with SBP and DBP was performed using 1,211 individuals (803 women, 408 men) derived from the population-based cohort across Estonia consisting of long-term blood donors not receiving any antihypertensive medication (Table 1). For binary analysis with essential hypertension, cases (*n* = 673) were defined as untreated subjects with BP readings ≥160/100 mmHg based on the median of several measurements or patients receiving antihypertensive therapy. Normotensive controls (*n* = 601; SBP ≤140 mmHg/DBP ≤90 mmHg) were selected from the population-based HYPEST cohort among the subjects that have never been prescribed antihypertensive treatment.

### Stage 2 Replication

In Stage 2, association testing of the *WNK1 AluYb8* insertion and BP was performed in two European samples—the BRIGHT (BRItish Genetics of HyperTension) and the CADCZ (Coronary Artery Disease in Czech)—and the results were combined in meta-analysis with Stage 1 study samples. The final sample size in meta-analysis with SBP and DBP was 3,494 subjects (2,088 women, 1,406 men; none treated with antihypertensive medication), and with hypertension 3,181 cases/2,720 controls (women, 1,851/1,692; men 1,330/1,028).

CADCZ study has been approved by the Ethics Committee of Charles University—First Faculty of Medicine (December 1996) and the BRIGHT study was approved by the Ethics Committee from local research committees of all partner institutes. All BRIGHT and CADCZ participants have given their written informed consent. The MRC British Genetics of Hypertension case–control samples have been recruited across the United Kingdom (http://www.brightstudy.ac.uk). Case ascertainment and phenotyping has been described elsewhere [Caulfield et al., 2003]. Briefly, cases originated from severely hypertensive families (1,700 sibpairs and 800 families collected for transmission disequilibrium test) were defined as patients under antihypertensive treatment and with BP readings ≥150/100 mmHg based on one reading or ≥145/95 mmHg based on the mean of three readings. Healthy normotensive controls (*n* = 2,000; BP <140/90 mmHg, no antihypertensive medication and no diagnosed diseases) were recruited by matching age, sex, and geographical distribution across the United Kingdom. Following the study design, the association analysis with SBP and DBP included healthy untreated BRIGHT controls (*n* = 1,639, 1,002 women, 637 men; Table 1). In case–control analysis hypertensives (*n* = 2,242) and normotensives (*n* = 1,639) were classified as defined at the recruitment. CADCZ subjects were recruited by the Cardiology Department of the Second Clinic of Internal Medicine, Faculty Hospital Královské Vinohrady in Prague Czech Republic, and details of the recruitment are published elsewhere [Janosikova et al., 2003]. Trained clinicians documented three measurements of resting BP and the median value was recorded. Association testing between SBP, DBP, and the carrier status of *WNK1 AluYb8* insertion included subjects not receiving antihypertensive treatment (*n* = 644, 283 women, 361 men; Table 1). Hypertensives (*n* = 266) and normotensives (*n* = 480) in case–control analysis were defined as in HYPEST.

### RNA Extraction and cDNA Synthesis

EDTA-blood (9 ml) was collected from nine female subjects from HYPEST study selected based on their alternative genotypes: three heterozygotes (Alu/−) and three homozygotes (Alu/Alu) for the *WNK1 AluYb8* insertion; and three subjects with wild-type (−/−) sequence (Supp. Table S5). Total RNA from leucocytes was extracted using LeukoLOCK™ Total RNA Isolation System (Ambion Inc., Austin, TX) including an optional TURBO™ DNase treatment to degrade the genomic DNA. Quantity and quality of extracted RNA was assessed with NanoDrop® ND-1000 UV-Vis Spectrophotometer (NanoDrop Technologies, LLC, Wilmington, DE). RNA was reverse transcribed using SuperScript™ III First-Strand Synthesis SuperMix for qRT-PCR (Life Technologies Corporation, Carlsbad, CA) according to the manufacturer's instructions (details in Supp. Text S1).

### Quantification of *WNK1* Transcripts by Real-Time PCR

**Figure 2:**
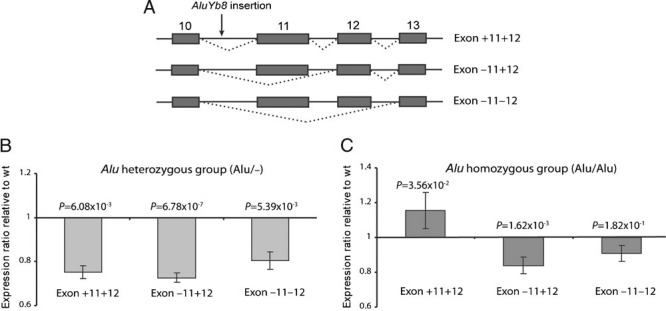
Expression of (**A**) three *WNK1* alternative splice-forms in blood leucocytes obtained from (**B**) heterozygous (Alu/−) and (**C**) homozygous (Alu/Alu) carriers of the *WNK1 AluYb8* insertion in comparison with the wild-type homozygote without the insertion. A: Alternative splicing of *WNK1* exons 10–13 is presented schematically according to Delaloy et al. [2003]. Black numbered boxes and horizontal lines represent exons and introns, respectively, and dotted lines indicate splicing events. B, C: Relative mRNA quantification of the targeted *WNK1* splice-forms in leucocytes was performed with real-time RT-PCR (Taqman assay, *HPRT* as a reference gene). Relative expression of each targeted *WNK1* splice-form in subjects with the *AluYb8* insertion (Alu/Alu homozygotes, Alu/− heterozygotes) is shown using the quantity of the transcript in wild-type homozygotes (−/−) as a reference value (wt = 1). The presented relative expression levels represent the mean values of the three study subjects within the genotype group (each individual represented by six data points from replicate experiments). Bars represent standard error of the relative expression. *P*-Values reflecting the differences between groups were estimated by Wilcoxon rank sum test.

Relative expression analysis of three *WNK1* splice forms (ex+11+12, ex−11+12, and ex−11−12; Fig. 2A) was performed with real-time PCR. Primer-probe mix of the *WNK1* transcript including exon 11 (ex+11+12; Hs01018312_m1, amplicon size 78 bp) and selected reference gene *HPRT1* [Human HPRT1 (HGPRT) Endogenous Control (VIC/MGB Probe, Primer Limited, amplicon size 100 bp)] were purchased from Applied Biosystems, Inc. (Foster City, CA). Primers and probes for the *WNK1* transcripts lacking exon 11 (ex−11+12) and both exons 11 and 12 (ex−11−12) were designed using Primer Express version 3.0 (Applied Biosystems Inc.). Oligonucleotide sequences are given in Supp. Table S4.

The real-time RT-PCRs were performed using Applied Biosystems 7900HT Fast Real-Time PCR system in 96 microwell plates. Target region and endogenous control were amplified in the same well. The experimental conditions for the real-time PCR are given in detail in Supp. Text S1. In total six replicate analyses of each of the nine extracted RNA samples were conducted: two independently synthesized cDNAs were assayed by RT-PCR reactions in triplicate.

### Statistical Analysis

Statistical differences in allele frequencies between populations were calculated using the Web-based Fisher's Exact Test calculator (http://www.langsrud.com/fisher.htm). The significance of the associations between the *WNK1 AluYb8* insertion and BP (SBP; DBP) as a quantitative trait was tested using linear regression (additive genetic model) with age and gender as covariates. Additive genetic model assumes a trend per copy of the minor allele to contribute to the trait or disease susceptibility on genotype categories. Association with the diagnosis of hypertension as a binary trait was assessed by logistic regression adjusted for age and sex. Association tests and calculation of LD between SNP pairs (*r*^2^) were implemented in the PLINK software, version 1.04 (http://pngu.mgh.harvard.edu/∼purcell/plink/). The Bonferroni threshold for multiple testing correction was estimated 0.05/9 = 5.56 × 10^−3^, taking into account the number of tested phenotypes (three) and tested study samples (three). Results were combined in a meta-analysis using the inverse-variance method under fixed-effects model using R, version 2.7.2 (R Development Core Team 2008, http://www.r-project.org/).

Normalized expression values of target regions were calculated using Microsoft® Excel®-based software Q-Gene [Muller et al., 2002]. Q-Gene calculates the normalized expression values of the target gene based on the Ct values and the reaction efficiencies of the target and the reference gene (here *HPRT*). For every study subject six replicate values of relative expression per each alternative *WNK1* splice form (ex+11+12, ex−11+12, and ex−11−12) was calculated. As each of the genotypes (Alu/Alu, Alu/−; −/−) was represented by three individuals, in total 18 data points were collected per transcript within a genotype group. The most outlier Ct value within the respective genotype group was excluded from the statistical testing. Differences of normalized expression values between alternative genotype groups were estimated by Wilcoxon rank sum test implemented in R software.

## Results

### Polymorphism Screening in *WNK1* and *WNK4* Conserved Noncoding Regions

DHPLC and/or DGGE assays were designed for screening novel polymorphisms in CNRs of the *WNK1* (29 targeted CNRs based on criteria outlined in Materials and Methods) and the *WNK4* (11 CNRs) genes (Supp. Table S1). Based on the in silico quality control criteria for assay design, nine regions were excluded from the wet-lab analysis. Finally, 31 CNRs entered variant detection in Eastern European essential hypertension patients (from HYPEST and CADCZ studies). Among the screened 31 CNRs, one SNP was identified in the *WNK4* and six SNPs in the *WNK1* gene (Supp. Table S6). All but one (rs36052085) of the detected SNPs were rare (minor allele frequency <10%), including three singletons (two novel). The functional effect of these SNPs was addressed neither by association study due to large sample size requirements nor by gene expression analysis due to unavailability of minor allele homozygotes.

In addition, in one of the *WNK1* CNRs, a novel unreported common indel (∼300 bp) was detected (Fig. 1; Supp. Table S6). Sequence analysis of this variant revealed a polymorphic insertion of an *AluYb8* element (288 bp without flanking T nucleotides) into a poly-T tract within *WNK1* intron 10,∼780 bp upstream from exon 11 (Supp. Fig. S2). This *Alu*-insertion was targeted to further evolutionary and population genetic analysis as well as was explored for the association with cardiovascular disease and the effect on the gene expression profile.

### *Alu* Distribution Among General Human Populations

The *WNK1 AluYb8* insertion was genotyped in 18 population samples from Europe, Asia, and Africa (Supp. Table S7 and Supp. Fig. S3). The genotyped variant was in Hardy-Weinberg equilibrium in all but one (Saharawi, *n* = 50) studied population samples. Frequency of the *AluYb8* insertion in human populations differed based on their geographic affiliation (Table 2, Supp. Fig. S3). The proportion of *WNK1 AluYb8* carriers in Sub-Saharan Africa was significantly lower (average allele frequency 4.8%; range: 2.1–7.0%) compared to North-African (mean: 16.4%, range: 10.4–25.0%; Fisher's Exact Test, *P* = 2.2 × 10^−6^), European (mean: 15.1%, range: 12.0–16.5%; *P* = 8.7 × 10^−9^), and Asian (mean: 15.9%, range: 11.6–22.0%; *P* = 9.4 × 10^−6^). On average, the allele frequency of the *WNK1 AluYb8* in Sub-Saharan Africa was ∼3.3 times lower than in other studied populations (*P* = 9.7 × 10^−9^).

**Table 2:** *WNK1* Intron 10 *AluYb8* Allele Frequencies in Population Groups

Group	No. of subjects	Allele frequency	Population composition
Eastern Europe	150	16.3%	Estonians, Czech
Western Europe	121	14.1%	CEPH/Utah families, Basques, Catalans
Gypsies	50	12.0%	Spanish Gypsies
Volga-Ural	94	17.0%	Tatars, Bashkirs
Eastern Asia	68	16.8%	Chinese Han, Koreans
North Africa	230	16.4%	Moroccans, Saharawi, Algerians, Tunisians
Sub-Saharan Africa	141	4.8%	Mandenkalu, Tanzanians, Gabon Bantus, Gabon Pygmies

### *Alu* Insertion in Primates and Conservation Around Insertion Site

The analysis of the *WNK1* intron 10 in 11 chimpanzees, 1 gorilla, and 1 orangutan revealed that the *WNK1 AluYb8* insertion has most probably occurred in human lineage. No *AluYb8* insertion was detected in the *WNK1* intron 10 of the studied primate genomes (Fig. 1, Supp. Fig. S1). Comparative sequencing of the *WNK1* genomic fragment (exon10/intron10/exon11) amplified from a chimpanzee and from human wild-type as well as *AluYb8* insertion carrying chromosomes revealed high conservation of intron 10 (Supp. Fig. S1). The substitution divergence between human wild-type and chimpanzee *WNK1* was 0, 0.2, and 1.1% for exon 10 (150 bp), exon 11 (459 bp), and intron 10 (1,211 bp), respectively. Overall substitution rate was 0.7 and 1.3% between *WNK1* exons and introns, respectively. The uniqueness of the *WNK1 AluYb8* insertion in human was supported by a negative result of the BLAST search among available genome sequences.

### Stage 1 Association Testing of the *WNK1 AluYb8* Insertion with Blood Pressure and Hypertension in the HYPEST Study

The identified *WNK1 AluYb8* insertion was tested for association with BP in the Estonian HYPEST cohort subjects (*n* = 1,211). The analysis detected significantly higher SBP (*P* = 1.26 × 10^−2^, effect 2.23 mmHg; linear regression, additive model) and DBP (*P* = 3.04 × 10^−2^, effect 1.22 mmHg; Table 3) among *Alu*-insertion carriers. Analysis in men and women separately revealed that the effect of the *AluYb8* insertion on BP might be female-specific (SBP: *P* = 1.32 × 10^−2^, effect 2.72 mmHg; DBP: *P* = 6.20 × 10^−3^, effect 1.84 mmHg), whereas no statistical support was found for the association in men (SBP, DBP, *P*>5.9 × 10^−1^). We also observed higher *WNK1 AluYb8* frequency among HYPEST essential hypertension patients (*n* = 673; 17.7%) compared to normotensive controls (*n* = 601; 14.5%; Supp. Table S8).

**Table 3:** Association of *AluYb8* Insertion with Systolic (SBP) and Diastolic (DBP) Blood Pressure

	HYPEST^a^ (*N* = 1,211/803/408)^d^		CADCZ^a^ (*N* = 644/283/361)		BRIGHT^b^ (*N* = 1,639/1,002/637)		Joint meta-analysis^c^ (*N* = 3,494/2,088/1,406)	
				
	Beta (SE)^e^	*P*-Value	Beta (SE)	*P*-Value	Beta (SE)	*P*-Value	Beta (SE)	*P*-Value
SBP								
All	2.23 (0.89)	**1.26 × 10^−2^**	0.05 (1.09)	9.65 × 10^−1^	1.01 (0.47)	**3.23 × 10^−2^**	1.12 (0.39)	4.03 × 10^−3^*^^
Women	2.72 (1.10)	**1.32 × 10^−2^**	2.45 (1.60)	1.26 × 10^−1^	1.09 (0.63)	8.24 × 10^−2^	1.59 (0.52)	1.99 × 10^−3^*^^
Men	0.78 (1.48)	5.97 × 10^−1^	−2.10 (1.48)	1.58 × 10^−1^	0.74 (0.68)	2.71 × 10^−1^	0.33 (0.57)	5.58 × 10^−1^
DBP								
All	1.22 (0.56)	**3.04 × 10^−2^**	0.10 (0.71)	8.95 × 10^−1^	0.60 (0.33)	7.22 × 10^−2^	0.67 (0.27)	**1.21 × 10^−2^**
Women	1.84 (0.67)	**6.19 × 10^−3^**	1.00 (1.07)	3.51 × 10^−1^	1.02 (0.44)	**2.00 × 10^−2^**	1.23 (0.35)	3.64 × 10^−4^*^^
Men	−0.17 (1.03)	8.67 × 10^−1^	−0.72 (0.94)	4.42 × 10^−1^	−0.20 (0.51)	6.95 × 10^−1^	−0.30 (0.41)	4.71 × 10^−1^

a Population-based subjects not receiving blood pressure-lowering medication.

b Normotensive controls across UK not receiving blood pressure-lowering medication.

c Meta-analysis of HYPEST, BRIGHT, and CADCZ; inverse-variance method under fixed-effect model.

d *N* = All/Women/Men.

e Linear regression (additive model, age, and gender as covariates) was used to test association with SBP and DBP (effect given as beta, SE). *P*<0.05 is given **in bold** and *P*-values resistant to Bonferroni correction for multiple testing are indicated with the asterisk (^*^). Bonferroni significance level was estimated α = 0.05/9 = 5.56 × 10^−3^ (3 phenotypes × 3 study samples). SBP, DBP, systolic and diastolic blood pressure; N, number of subjects; SE, standard error.

### Stage 2 Association Testing in BRIGHT and CADCZ, and Meta-Analysis Across Three European Sample Sets

To confirm the discovery association between the *WNK1 AluYb8* insertion and BP identified in the HYPEST study (Estonians), we performed Stage 2 replication testing in two independent European samples: the BRIGHT (the British) and the CADCZ (Czech) (Table 1). Stage 1 and Stage 2 results were combined in a joint meta-analysis (Table 3). Meta-analysis across all studies (*n* = 3,494) improved significantly the support for the association with BP (SBP, *P* = 4.03 × 10^−3^, effect 1.12 mmHg; DBP *P* = 1.21 × 10^−2^ effect 0.67 mmHg). The pronounced effect of this *Alu-*insertion on BP in women was confirmed (*n* = 2,088; SBP, *P* = 1.99 × 10^−3^, effect 1.59 mmHg; DBP *P* = 3.64 × 10^−4^ effect 1.23 mmHg). Detected associations of the *WNK1 AluYb8* insertion with SBP in the full sample and with female SBP and DBP remained significant after stringent correction for multiple testing (Bonferroni threshold α = 0.05/9 = 5.56 × 10^−3^; Table 3). Consistent with the discovery sample, no support was detected to the effect of *AluYb8* insertion on male BP (*n* = 1,406; SBP, DBP, *P*>4.7 × 10^−1^).

Consistent with HYPEST, the trend for higher frequency of *WNK1 AluYb8* was observed in CADCZ hypertensive patients compared to controls (17.1 vs. 15.3%), but not in the BRIGHT cases representing extreme family based hypertension (Supp. Table S8).

### Linkage Disequilibrium (LD) Landscape Between the Novel *AluYb8* Insertion and Reported Blood Pressure-Associated *WNK1* SNPs in HYPEST and BRIGHT

When allelic association was assessed between the *AluYb8* insertion and rs765250 the associated SNP with SBP variation in a meta-analyses including the BRIGHT study and the HYPEST subjects in a previous report [Newhouse et al., 2009], low LD was estimated in both samples (HYPEST: *r*^2^ = 0.069; BRIGHT: *r*^2^ = 0.080). However, *AluYb8* was in strong LD with three previously genotyped *WNK1* SNPs in the BRIGHT resource: rs11064527 (*r*^2^ = 0.821; MAF = 0.16, intron 1), rs12816718 (*r*^2^ = 0.921; MAF = 0.15, intron 6), and rs956868 (*r*^2^ = 0.975; MAF = 0.14, exon 13) (Supp. Table S9). Amino acid alignment of the *WNK1* exon 13 among vertebrates exhibited low evolutionary conservation at the position of rs956868 coding for two alternative amino acids Proline or Threonine in human (Supp. Fig. S4). When association testing with BP was performed for the BRIGHT individuals genotyped for the *AluYb8* insertion as well as the three SNPs in LD (*n* = 1,421), the strongest association was detected with *AluYb8* (Supp. Table S10).

### The Impact of the *AluYb8* Insertion on the Expression Profile of *WNK1* in Leucocytes

The human *WNK1* gene codes for a high number of mRNA transcripts and extensive alternative splicing has been described for exons 9, 11, and 12 [Verissimo and Jordan, 2001]. The major human *WNK1* transcript completely lacks exons 11 and 12 [Delaloy et al., 2003]. In order to explore the functional effect of the *AluYb8* insertion on the expression profile of *WNK1* alternative transcripts, we quantified the gene transcripts in mRNA extracted from the leucocytes of nine women with alternative genotypes (Supp. Table S5). The study subjects included three individuals heterozygous and three homozygous for the *AluYb8* insertion, as well as three wild-type genotype carriers. The expression of three *WNK1* splice forms was addressed using relative quantification method based on real-time RT-PCR assays. The studied splice forms differed by alternative inclusion/exclusion of exon 11 and exon 12 (Fig. 2A). Compared to the subjects with the wild-type genotype, the heterozygous *AluYb8* carriers had significantly lower expression of all three splice-forms (Wilcoxon rank sum test; ex+11+12: *P* = 6.08 × 10^−3^; ex−11+12: *P* = 6.78 × 10^−7^; ex−11−12: *P* = 5.39 × 10^−3^; Fig. 2B). Consistently, homozygous *AluYb8* carriers showed lower expression level of splice forms ex−11+12 (*P* = 1.62 × 10^−3^) and ex−11−12 (*P* = 1.82 × 10^−1^; Fig. 2C). However, the expression of the full-length *WNK1* transcript, which includes both exon 11 and 12 (ex+11+12) was upregulated among *AluYb8* −homozygous carriers (*P* = 3.56 × 10^−2^). We conclude that the carrier status of the *AluYb8* insertion may have an impact on the profile in *WNK1* transcript in leucocytes*.*

## Discussion

We targeted conserved noncoding regions in hypertension candidate genes *WNK1* and *WNK4* to polymorphism screening in order to identify functional variants potentially contributing to BP determination. We identified a novel human-specific polymorphic *AluYb8* insertion in *WNK1* intron 10. The *AluYb8* insertion belongs to a young *Alu* subfamily represented with ∼2,200 copies in the human genome compared to only nine insertions detected in chimpanzee [Gibbons et al., 2004]. Consistently, we were unable to detect the studied *WNK1 AluYb8* insertion in chimpanzee, gorilla, and orangutan (Fig. 1). As *AluYb8* elements are relatively mobile *Alu* repeats, they represent together with *AluYa5* subfamily ∼58% of the polymorphic *Alu-*s in the human genome [Bennett et al., 2008]. The increased carrier frequency of *WNK1 AluYb8* insertion out of Africa is consistent with recent studies showing that the allele frequencies of polymorphic *Alu* insertions tend to be lowest in Sub-Saharan populations (Table 2) [Watkins et al., 2001, 2003]. In Africa, the fraction of carriers of polymorphic *Alu*-s increase with sharp cline in the north of Sahara compared to the populations living south of the desert [Comas et al., 2000].

Our study identified a significant association between *WNK1 AluYb8* insertion and BP in the Estonian HYPEST cohort and confirmed this finding in the meta-analysis across three independent European study samples (*n* = 3,494; HYPEST, BRIGHT, CADCZ). The carriers of the *WNK1 Alu* insertion had a consistent tendency for higher blood pressure (Table 3). Notably, when the analysis was performed using samples stratified by gender, the *WNK1 AluYb8* insertion was associated with BP only among women (meta-analysis: SBP, *P* = 1.99 × 10^−3^; DBP, *P* = 3.64 × 10^−4^) and no association was detected in men. Consistent with our findings, a sex-specific effect on BP determination was recently shown for a *WNK1* SNP in intron 1 (rs10774461), which was also associated with BP only in females [Padmanabhan et al., 2010]. Similar gender-specific effects have been reported for the polymorphic *Alu* insertion (rs4646994) located in intron 16 of the *ACE* (*angiotensin converting enzyme*) gene [Rigat et al., 1990]. Three independent studies showed that *ACE Alu* I/D variant is associated with the hypertension risk only in men and not in women [Higaki et al., 2000; O'Donnell et al., 1998; Stankovic et al., 2002]. These differential effects on BP may reflect the differences in male and female physiology. Sex hormones have an important role in regulating a variety of renal transport functions and may contribute to gender differences in several kidney-related traits. Clinical observations in humans and experimental animals have shown that renal structure and functions under various physiological, pharmacological, and toxicological conditions are different in males and females, and that these differences may be related to the sex-hormone regulated expression and action of transporters in epithelial cells of nephrons [reviewed by Sabolic et al., 2007].

Previously, BP variation in the BRIGHT and the HYPEST subjects has been associated with the *WNK1* SNP rs765250 [Newhouse et al., 2009]. We detected low allelic association between *AluYb8* and rs765250 in both samples, which may indicate independent effects of *WNK1* intron 1 and intron 10 polymorphisms on BP. Interestingly, strong LD (*r*^2^>0.8) between *AluYb8* and three previously studied *WNK1* SNPs in the BRIGHT study alone (rs11064527, rs12816718, rs956868) positioned *AluYb8* on the *WNK1* haplotype, was reported to show borderline associations with SBP and DBP (*P*≤9 × 10^−2^) and a strong association with 24-hr urine potassium (*P*<1 × 10^−4^) [Newhouse et al., 2009]. Although this haplotype includes a nonsynonymous change (rs956868; Exon 13, Thr1316Pro, NP_001171914), *P*-values for the association with BP were the lowest for the *AluYb8* insertion compared to SNPs in LD. Previously, rs956868 has been shown to exhibit suggestive effect on ambulatory SBP in Europeans (*P*<9 × 10^−2^) [Tobin et al., 2005], and a significant association with SBP in Japanese (*P*<5 × 10^−2^) [Osada et al., 2009]. The current and previous studies consistently report the highest BP levels in homozygotes for the minor allele of these polymorphisms (rs956868: Thr/Thr in LD with *AluYb8*: +/+). Functional assays should bring understanding whether the detected association with BP is driven by one primary variant or by a combinatory effect of the haplotype-forming alleles.

The design of the current study did not allow us to draw any conclusion about the contribution of the *WNK1 AluYb8* insertion to the risk for developing hypertension. The initially observed but not confirmed higher proportion of *AluYb8* carriers among hypertensives may have resulted from nonoptimal selection of HYPEST controls (biased to too young), and/or different recruitment strategies of hypertensive patients among studies (HYPEST, essential hypertension; BRIGHT, extreme family-based hypertension; CADCZ, hypertension in CAD patients).

Although a majority of *Alu* elements are considered to be neutral residents of the human genome, an inserted copy of an *Alu* repeat could interrupt structurally or functionally important genomic regions and consequently affect the expression of a locus [Batzer and Deininger, 2002; Callinan and Batzer, 2006]. *Alu* elements may alter gene expression through modulating alternative splicing, RNA editing, epigenetic regulation, and translation regulation [Cordaux and Batzer, 2009; Hasler and Strub, 2006]. So far, 33 diseases directly caused by novel *Alu* insertions have been identified [Belancio et al., 2008]. Our study using mRNA extracted from human leucocytes indicated a potential effect of the presence of the *AluYb8* insertion in *WNK1* intron 10 on the expressional profile of *WNK1* alternative transcripts. Splicing is an incompletely understood process carried out by large macromolecular complex spliceosome and directed by numerous regulatory elements located within exonic and intronic sequence [Black, 2003]. The size of the *WNK1* intron 10 (human wild-type 1,211 bp) is remarkably increased by the ∼300 bp *AluYb8* insertion (human variant >1,500 bp). Thus, we hypothesize that the presence of the *AluYb8* insertion may disrupt the spatial intronic structure and/or disarrange the possible splicing regulatory sequences within *WNK* intron 10. Consequently, it may affect the splicing efficiency of the down-stream exons 11 and 12. As alternative splicing tends to be a tissue and developmental stage specific process [Xu et al., 2002], the impact of *AluYb8* insertion on the expressional profile of *WNK1* may vary in different tissues. The current study design was limited to addressing the effect of *AluYb8* insertion on *WNK1* expressional profile in leucocytes using a small number of samples. Further in vitro and in vivo studies using renal tissues would reveal the potential effect of this *Alu*-insertion on *WNK1* expressional profile in kidneys, where it plays an important role in contributing to the regulation of ion transport.

In summary, we identified a novel human-specific polymorphic *AluYb8* insertion in *WNK1*. This *AluYb8* insertion showed significant replicated association with blood pressure and a potential effect on the expressional profile of alternative *WNK1* transcripts in leucocytes.
