# Supplementary material for: Novel polymorphic AluYb8 insertion in the WNK1 gene is associated with blood pressure variation in Europeans
Source: Hum Mutat. 2011 Apr 21;32(7):806–14. doi: 10.1002/humu.21508 (PMC3298642; doi:10.1002/humu.21508)
Supplement: Supplementary file 1 [file humu0032-0806-SD1.pdf]

SUPPORTING INFORMATION

Novel polymorphic *AluYb8* insertion in the *WNK1* gene is associated with blood pressure variation in Europeans

Margus Putku[1], Katrin Kepp[1], Elin Org[1], Siim Sõber[1], David Comas[2], Margus Viigimaa[3,4], Gudrun Veldre[1,5], Peeter Juhanson[1], Pille Hallast[1], Neeme Tõnisson[1], HYPEST†, Sue Shaw-Hawkins[6], Mark J. Caulfield[6], BRIGHT†, Elza Khusnutdinova[7], Viktor Kožich[8], Patricia B. Munroe[6], Maris Laan[1]*

[1]Human Molecular Genetics Research Group, Institute of Molecular and Cell Biology, University of Tartu, Tartu, Estonia

[2]Institute of Evolutionary Biology (UPF-CSIC), CEXS-UPF-PRBB, Universitat Pompeu Fabra, Barcelona, Spain

[3]Centre of Cardiology, North Estonia Medical Centre, Tallinn, Estonia

[4]Tallinn University of Technology , Department of Biomedical Engineering, Chair of Medical Physics, Tallinn, Estonia

[5]Department of Cardiology, University of Tartu, Tartu, Estonia

[6]Clinical Pharmacology and The Genome Centre, William Harvey Research Institute, Barts and The London School of Medicine and Dentistry, Queen Mary University of London, London EC1M 6BQ

[7]Institute of Biochemistry and Genetics, Ufa Science Center, Russian Academy of Sciences, Ufa, Bashkortostan, Russia

[8]Institute of Inherited Metabolic Diseases, Charles University – First Faculty of Medicine, Prague, Czech Republic

†HYPertension in ESTonia (HYPEST), the British Genetics of Hypertension (BRIGHT); additional BRIGHT consortium contributors are given in Supp. Text S1.

* Correspondence and reprint requests to: Maris Laan, Institute of Molecular and Cell Biology, University of Tartu, Riia 23, 51010 Tartu, Estonia; Telephone: +372-7375008, Fax: +372-7420286, Email: maris.laan@ut.ee

LIST OF SUPPORTING INFORMATION:

Supp. Text S1. List of additional BRIGHT consortium contributors, additional information for Materials and Methods

Supp. Figure S1. PCR amplification of the locus for *WNK1* intron 10 *AluYb8* insertion in eleven chimpanzees as resolved by agarose gel (3%) electrophoresis.

Supp. Figure S2. Sequence alignment of the *WNK1* genomic fragment spanning from exon 10 to exon 11.

Supp. Figure S3. Global map of the allele frequencies of the *WNK1 AluYb8* insertion in studied human population samples from Eurasia and Africa.

Supp. Figure S4. Amino acid alignment of the *WNK1* exon 13 among vertebrates.

Supp. Table S1. Characteristics of the *WNK1* (12p13.33; Ensembl ID: ENSG00000060237) and *WNK4* (17q21.31; Ensembl ID: ENSG00000126562) non-coding regions selected for polymorphism screening.

Supp. Table S2. Polymorphism screening primers for DHPLC

Supp. Table S3. Polymorphism screening primers for DGGE

Supp. Table S4. Sequences of primers and probes used in PCR and RT-PCR

Supp. Table S5. Characteristics of the individuals used for *WNK1* expression profiling in leucocytes

Supp. Table S6. Polymorphisms detected in screened conserved non-coding regions of human *WNK1* and *WNK4* genes

Supp. Table S7. Allele frequencies of the *WNK1 AluYb8* insertion in general human population samples

Supp. Table S8. Association testing of the *WNK1* intron 10 *AluYb8* with hypertension

Supp. Table S9. Linkage disequilibrium (r^2) between the *WNK1 AluYb8* and common SNPs in the *WNK1* gene previously reported in the BRIGHT study

Supp. Table S10. Association of the *WNK1 AluYb8* and SNPs rs11064527, rs12816718, rs956868 with blood pressure in the BRIGHT

Supp. Text S1. List of additional BRIGHT consortium contributors, additional information for Materials and Methods

Additional MRC the British Genetics of Hypertension (BRIGHT) study consortium members:

Toby Johnson, Stephen J. Newhouse, Philip J. Howard, Abiodun Onipinla, Anna Dominiczak, Nilesh J. Samani, Martin Farrall, Morris Brown, Mark Lathrop, John Connell

Affiliations:

Clinical Pharmacology and Barts and the London Genome Centre, William Harvey Research Institute, Barts and the London School of Medicine, Queen Mary University of London, London, UK (T.J., S.J.N., P.J.H, A. O.); BHF Glasgow Cardiovascular Research Centre, Division of Cardiovascular and Medical Sciences, University of Glasgow, Western Infirmary, Glasgow, UK (A.D.); Department of Cardiovascular Sciences, University of Leicester, Glenfield Hospital, Leicester, UK (N.S.); Department of Cardiovascular Medicine, University of Oxford, Wellcome Trust Centre for Human Genetics, Oxford, UK (M.F.); Clinical Pharmacology and the Cambridge Institute of Medical Research, University of Cambridge, Addenbrooke's Hospital, Cambridge, UK (M.B.); Centre National de Genotypage, Evry, France (M.L.); University of Dundee, Medical School, Ninewells Hospital and Medical School Dundee, UK (J.C.)

Details of the design and experimental condition of the DGGE and DHLPC assays

DGGE primers with GC rich tail at 5′ end of one primer of the primer pair were designed with program MELTingeny 1.0.1 using primer design criteria recommended by Ingeny International. Supplementing the 5' end of the reverse primer with a GC clamp (a sequence of 40 G and C deoxynucleotides) allowed the detection of single-base substitutions present in the fragment.

DHPLC primers were designed according to manufacturer recommendations (DHPLC; Wave Technologies Inc. USA) using web-based program Primer3 (3). The calculations of the appropriate melting conditions for each of the selected DHPLC PCR fragments we used web based DHPLC Melt Program (http://insertion.stanford.edu/melt.html). The uniqueness of the primer sequence in the human genome were tested and verifying using GenomeTester1.3 (http://bioinfo.ut.ee/genometester/). The DGGE and DHPLC primers are given in Supp. Table S1.

Regions targeted for polymorphism screening were amplified by touchdown method using 100 ng genomic DNA, Smart-Taq Hot DNA polymerase (Naxo OÜ, Tartu, Estonia), GeneAmp PCR System 2700 thermal cycler (Applied Biosystems Inc., USA) and amplification conditions described elsewhere (1). DGGE electrophoresis by INGENY PhorU2 system was performed according to the manufacturer's instructions (Ingeny, Goes, Netherlands). Electrophoresis (16-17 hours) was carried out at 58ºC, amperage of 40 mA and voltage of 100 V. The DHPLC analysis was performed according to the manufacturer's recommendations (Wave Technologies Inc. USA) using pools consisting of the genomic DNA from three individuals. Individual DNA probes exhibiting unusual pattern in the DGGE analysis or forming a DNA-pool indicative to the presence of a polymorphism in the DHPLC analysis were resequenced to determine the exact changes in the genomic sequence.

Recruitment of individuals to the HYPEST study

The HYPEST (n=1823) study has been approved by the Ethics Committee on Human Research of University of Tartu, Estonia. All of the study participants are of Eastern European ancestry,

have filled a self-administrated epidemiological questionnaire recording to their past and present health and life-style and have given their written informed consent.

The HYPEST sample collection represents a case-cohort study. Essential hypertension patients were recruited by blood pressure specialists during the patients' ambulatory visits or hospitalization at the North Estonia Medical Center, Centre of Cardiology, or at the Cardiology Clinic, Tartu University Hospital, Estonia. The HYPEST healthy control cohort was recruited from among the long-term blood donors across Estonia. All the included donors had no personal history of cardiovascular diseases and had also never been prescribed any relevant medications.

Details of the cDNA synthesis protocol

Total RNA from leucocytes was extracted using LeukoLOCK™ Total RNA Isolation System (Ambion Inc, Austin, Texas, USA) including an optional TURBO™ DNase treatment to degrade genomic DNA. Concentration of extracted RNA was measured with NanoDrop® ND-1000 UV-Vis Spectrophotometer (NanoDrop Technologies, LLC, Wilmington, Delaware, USA).

RNA was reverse transcribed using SuperScript™ III First-Strand Synthesis SuperMix for qRT-PCR (Life Technologies Corporation, Carlsbad, California, USA) in the final volume of 20 µl containing 0,4 µg of RNA, 10 µl of 2X RT Reaction Mix [oligo$(dT)_{20}$ (2.5 µM), random hexamers (2.5 ng/µl), 10 mM MgCl2, and dNTPs], 2µl of RT Enzyme Mix (includes SuperScript™ III RT and RNaseOUT™) and DEPC-treated water. The reaction mixture was incubated at 25ºC for 10 minutes and at 50ºC for 30 minutes. Reaction was terminated at 85ºC at 5 minutes and chilled on ice. cDNA was treated with 2 U of *E.coli* Rnase H at 37ºC for 20 minutes to remove the RNA template from the cDNA:RNA hybrid molecule.

Detailed conditions of the real-time RT-PCR reactions

The 20 µl PCR reactions consisted of 2 µl cDNA product 4X dilution, 4 µl of 5X HOT FIREPol® Probe qPCR Mix (Solis BioDyne, Tartu, Estonia) containing HOT FIREPol® DNA Polymerase, 5X Probe qPCR buffer, 15 mM $MgCl_2$, dNTPs and 2,5 µM ROX dye, as well as primers and a probe in following concentration: 400 nM forward and reverse primer, 250 nM probe for spliced transcripts or 1 µl of 20X primer-probe mix for transcript including exon 11 (Hs01018312_m1) and 0.9 µl (ex+11+12 and ex−11−12) or 0.8 µl (ex−11+12) of 20X ready-to-use primer-probe mix for endogenous control *HPRT1*. The PCR protocol was identical in all runs: incubation step at 95ºC for 15' followed by 40 two-step amplification cycles (15'' at 95ºC; 1' at 60ºC).

References

Hallast P, Nagirnaja L, Margus T, and Laan M. 2005. Segmental duplications and gene conversion: Human luteinizing hormone/chorionic gonadotropin beta gene cluster. *Genome Res* 15: 1535-1546.

Rozen S, and Skaletsky H. 2000. Primer3 on the WWW for general users and for biologist programmers. *Methods Mol Biol* 132: 365-386.

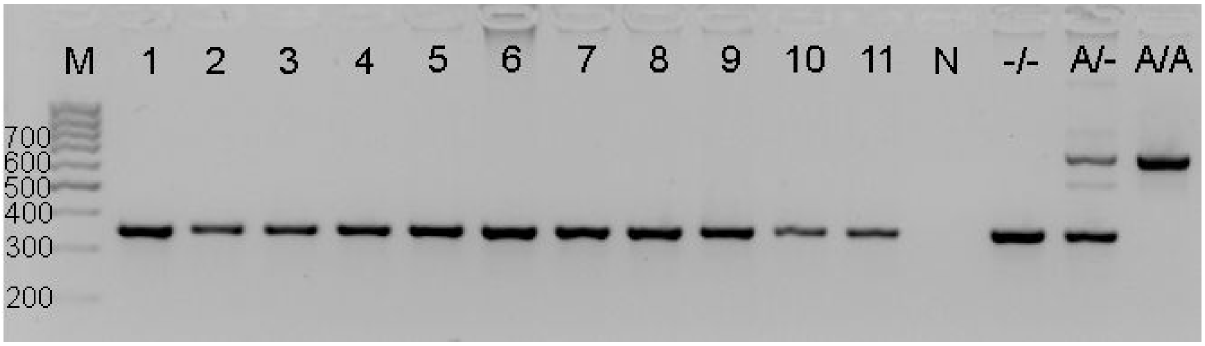

Supp. Figure S1. PCR amplification of the locus for *WNK1* intron 10 *AluYb8* insertion in eleven chimpanzees as resolved by agarose gel (3%) electrophoresis. In all analyzed individuals (numbered 1-11) the resulting PCR product corresponded to the wild-type genotype lacking the *WNK1* intron 10 *AluYb8* insertion. Human genomic DNAs representing alternative genotypes were used as positive controls: wild-type genotype without *AluYb8* insertion (–/–, PCR product 353 bp); heterozygous (A/–) and homozygous (A/A, PCR product 660 bp) carriers of the insertion.

N, negative control; M, marker (Fermentas MassRuler™ DNA Ladder, Low Range)

Supp. Figure S2. Sequence alignment of the *WNK1* genomic fragment spanning from exon 10 to exon 11. Human chromosomes carrying (Alu +) and not carrying (Alu –) the *AluYb8* insertion are shown in comparison with the respective genomic region in common chimpanzee (*Pan troglodytes*). The sequences of the *WNK1* exons 10 and 11 are shown in blue and the *AluYb8* insertion to human *WNK1* intron 10 in red font. The substitution-based divergence between human wild-type (Alu –) and chimpanzee sequences (excluding species-specific insertion/deletions) is 0%, 0.2% and 1.1% for exon 10 (150 bp), exon 11 (459 bp) and intron 10 (1211 bp), respectively. Sequence alignment was performed using the web-based global alignment program ClustalW2 (http://www.ebi.ac.uk/Tools/clustalw2/).

EXON 10:

Human Alu (+)
CAGCAGGGAATACAGCAGACAGCCCCTCCTCAACAGACAGTGCAGTATTCACTTTCACAG 60
Human Alu (–)
CAGCAGGGAATACAGCAGACAGCCCCTCCTCAACAGACAGTGCAGTATTCACTTTCACAG 60
Chimpanzee
CAGCAGGGAATACAGCAGACAGCCCCTCCTCAACAGACAGTGCAGTATTCACTTTCACAG 60

Human Alu (+)
ACATCAACCTCCAGTGAGGCCACTACTGCACAGCCAGTGAGTCAACCTCAAGCTCCACAA 120
Human Alu (–)
ACATCAACCTCCAGTGAGGCCACTACTGCACAGCCAGTGAGTCAACCTCAAGCTCCACAA 120
Chimpanzee
ACATCAACCTCCAGTGAGGCCACTACTGCACAGCCAGTGAGTCAACCTCAAGCTCCACAA 120

Human Alu (+) **INTRON 10**
GTCTTGCCTCAAGTATCAGCTGGAAAACAG GTAAACTTTTTTTTTTTTTTTAAACAGGTA 180
Human Alu (–)
GTCTTGCCTCAAGTATCAGCTGGAAAACAG GTAAACTTTTTTTTTTTTTTTAAACAGGTA 180
Chimpanzee
GTCTTGCCTCAAGTATCAGCTGGAAAACAG GTAAACTTTTTTTTTTTTTTTAAACAGGTA 180

Human Alu (+)
AACTCTTAATTTCTGAAAGGGTGCTAAAAGGGATTTCCATGTAACTTGTCCTTTCATGTG 240
Human Alu (–)
AACTCTTAATTTCTGAAAGGGTGCTAAAAGGGATTTCCATGTAACTTGTCCTTTCATGTG 240
Chimpanzee
AACTCTTAATTTCTGAAAGGGTGCTAAAAGGGATTTCCATGTAACTTGTCCTTTCATGTG 240

Human Alu (+)
GATAGACTTCTACCTTTTCTTCTAAGGGTAACCAACCCTTGAAGTAGGTTAATCTCATTG 300
Human Alu (–)
GATAGACTTCTACCTTTTCTTCTAAGGGTAACCAACCCTTGAAGTAGGTTAATCTCATTG 300
Chimpanzee
GATAGACTTCTACCTTTTCTTCTAAGGGTAACCAACCCTTGAAGTAGGTTAATCTCATTG 300

Human Alu (+)
CAGAAATGAAGTGAAGATCACCTCATTGGTGCATATGCATTATTTAATGTAAATGGGTAC 360
Human Alu (–)
CAGAAATGAAGTGAAGATCACCTCATTGGTGCATATGCATTATTTAATGTAAATGGGTAC 360
Chimpanzee
CAGAAATGAAGTGAAGATCACCTCATTGGTGCATATGCATTATTTAATGTAAATGGGTAC 360

Human Alu (+)
GTTACTGACAGCAGTGACAATCCAAAGTTTCACTTCTAGTCTTACCAGTCCAAAATTGAT 420
Human Alu (–)
GTTACTGACAGCAGTGACAATCCAAAGTTTCACTTCTAGTCTTACCAGTCCAAAATTGAT 420
Chimpanzee
GTTACTGACAGCAGTGACAATCCAAAGTTTCACTTCTAGTCTTACCAGTCCAAAATCGAT 420

Human Alu (+)
GTAACAGGTATTAGAAATAACAGGTTAAATAAAGGTGCTCTGGTAACTGAACCTATGACA 480
Human Alu (–)
GTAACAGGTATTAGAAATAACAGGTTAAATAAAGGTGCTCTGGTAACTGAACCTATGACA 480
Chimpanzee
TTAACAGGTATTAGAAATAACAGGTTAAATAAAGGTGCTCTGGTAACTGAACCTATGACA 480

Human Alu (+)
TTCCCTAGAATTGTGTCAGCTATGTCTGATATTCTAGGTTTGAAACTATGCTTTATTTTC 540
Human Alu (–)
TTCCCTAGAATTGTGTCAGCTATGTCTGATATTCTAGGTTTGAAACTATGCTTTATTTTC 540
Chimpanzee
TTCCCTAGAATTGTGTCAGCTATGTCTGATATTCTAGGTTTGAAACTATGCTTTATTTTC 540

Human Alu (+) ***AluYb8* insertion**
ATGTAGATTTCTGCACTGTTTACTGTGTGGAATGTTTTTTTTTTTTTTTTTT GAGACG 600
Human Alu (–)
ATGTAGATTTCTGCACTGTTTACTGTGTGGAATGTTTTTTTTT- - - - - - - - - - - - - - - - - 583
Chimpanzee
ATGTAGATTTCTGCACTGTTTACTGTGTGGAATGTTTTTTTTTT- - - - - - - - - - - - - - - - - 584

Human Alu (+)
GAGTCTCGCTCTGTCGCCCAGGTCGGACTGCGGACTGCAGTGGCGCAATCTCGGCTCACT 660
Human Alu (–)
- -
Chimpanzee
- -

Human Alu (+)
GCAAGCTCCGCTTCCCGGGTTCACGCCATTCTCCTGCCTCAGCCTCCCGAGTAGCTGGGA 720
Human Alu (–)
- -
Chimpanzee
- -

Human Alu (+)
CTACAGGCGCCCGCCACCGCGCCCGGCTAATTTTTTGTATTTTTAGTAGAGACGGGGTTT 780
Human Alu (–)
- -
Chimpanzee
- -

Human Alu (+)
CACCTTGTTAGCCAGGATGGTCTCGATCTCCTGACCTCATGATCCACCCGCCTCGGCCTC 840
Human Alu (–)
- -
Chimpanzee
- -

Human Alu (+)
CCAAAGTGCTGGGATTACAGGCGTGAGCCACCGCGCCCGGCC TTTTTTTCTTAAGTAGT 900
Human Alu (–)
- CTTAAGTAGT 593
Chimpanzee
- CTTAAGTAGT 594

Human Alu (+)
CCTCCTAATCACTTGAGAAGTACCCTGTAGAAGGTATGCTTTTCTGAATGACCTTTTTTC 960
Human Alu (–)
CCTCCTAATCACTTGAGAAGTACCCTGTAGAAGGTATGCTTTTCTGAATGACCTTTTTTC 653
Chimpanzee
CCTCCTAATCACTTGAGAAGTACCCTGTAGAAGTTATGCTTTTCTGAATGACCTTTGTTC 654

Human Alu (+)
AACTTTATTTTTGAGTACTGTTGTGCATGTATGTGCACAGTGTATTGTTACCCTAATATT 1020
Human Alu (–)
AACTTTATTTTTGAGTACTGTTGTGCATGTATGTGCACAGTGTATTGTTACCCTAATATT 713
Chimpanzee
AACTTTATTTTTGAGCACTGTTGTGCATGTATGTGCACAGTGTATTGTTACCCTAATATT 714

Human Alu (+)
TCATCACATCTGAGATGCTATCAACTTTAAAATGTACCATATTTTATGTACCACATTATC 1080
Human Alu (–)
TCATCACATCTGAGATGCTATCAACTTTAAAATGTACCATATTTTATGTACCACATTATC 773
Chimpanzee
TCATCACATCTGAGATGCTATCAACTTTAAAATGTACCA- - - - - - - - - - - - - CATTATC 760

Human Alu (+)
AGTTATAAAGTAGATCCCAATTTCTCACATGTTAAAATGATCAGGTAGTGGAGAGGGATG 1140
Human Alu (–)
AGTTATAAAGTAGATCCCAATTTCTCACATGTTAAAATGATCAGGTAGTGGAGAGGGATG 833
Chimpanzee
AGTTATAAGGTAGATCCCAATTTCTCACATGTTAAAATGGTCAGGTAGTGGAGAGGGATG 820

Human Alu (+)
ATTGCATCCTAGAATGAGTGAAATATGTGAATTATACCAATTTTTATTGAGCCATGTTGG 1200
Human Alu (–)
ATTGCATCCTAGAATGAGTGAAATATGTGAATTATACCAATTTTTATTGAGCCATGTTGG 893
Chimpanzee
ATTGCATCCTAGAATGAGTGAAATATGTGAATTATACCAATTTTTATTGAGCCATGTTGG 880

Human Alu (+)
ACTTAAGAATTTTAGAAATAATACGAATGAAATCTTAATTCCACTGGGAAGGAATTCATT 1260
Human Alu (–)
ACTTAAGAATTTTAGAAATAATACGAATGAAATCTTAATTCCACTGGGAAGGAATTCATT 953
Chimpanzee
ACTTAAGAATTTTAGAAATAATATGAATGAAATCTTAATTCCACTGGGAAGGAATTCATT 940

Human Alu (+)
TGAATTTCAGTAACTACTGTAACAGCAGTCGTAACTTTAGTGATAGCATTATGGTATATA 1320
Human Alu (–)
TGAATTTCAGTAACTACTGTAACAGCAGTCGTAACTTTAGTGATAGCATTATGGTATATA 1013
Chimpanzee
TGAATTTCAGTAACTACTGTAATAGCAGTCGTAACTTTAGTGATAGCATTATGGTATATA 1000

Human Alu (+)
AAATATATTCCAATAAAGCTGTTAAAAAAAAAA-GCCAACCCCTTGTCTATGGAAGGGTC 1379
Human Alu (–)
AAATATATTCCAATAAAGCTGTTAAAAAAAAAA-GCCAACCCCTTGTCTATGGAAGGGTC 1072
Chimpanzee
AAATATATTCCAATAAGGCTGTAAAAAAAAAAAAGCCAACCCCTTGTCTATGGAAGGGTC 1060

Human Alu (+)
CTCTTCTATTGCCAAATGCTGAAGCATTAGCAAATATTTCTATGACAAAAGGTGTAGAAC 1439
Human Alu (–)
CTCTTCTATTGCCAAATGCTGAAGCATTAGCAAATATTTCTATGACAAAAGGTGTAGAAC 1132
Chimpanzee
CTCTTCTATTGCCAAATGCTGAAGCATTAGCAAATATTTCTATGACAAAAGGTGTAGAAC 1120

Human Alu (+)
AGTAATAGTCTATTTAGCCTCTTTCTCTCCTGCTCTCCTTTCCATATTTTTATGTGGCAT 1499
Human Alu (–)
AGTAATAGTCTATTTAGCCTCTTTCTCTCCTGCTCTCCTTTCCATATTCTTATGTGGCAT 1192
Chimpanzee AGTAATAGTCTATTTAGCCTCTTTCTCTCCTGCTCTCCTTTCCATATTCTTATGTGGCAT 1180

Human Alu (+)
ATTAACTTAACACTAATGTATGCAGGGTTTTGTTGGTTTGGTGTTTTTTTTTTTTTGTTT 1559
Human Alu (–)
ATTAACTTAACACTAATGTATGCAGGGTTTTGTTGGTTTGGTGTTTTTTTTTTT- - GTTT 1250
Chimpanzee
ATTAACTTAACACTAATGTATGCAGGGTTTTGTTGGTTTGGTGTTTTTTTTGTT- - - - TT 1236

Human Alu (+)
GTTTTTTCCTTCTTTTTGGCTAATACATAAATCTTGCTTTTGGCAGCCTTGCTTTTTTTT 1619
Human Alu (–)
GTTTTTTCCTTCTTTTTGGCTAATACATAAATCTTGCTTTTGGCAGCCTTGCTTTTTTTT 1310
Chimpanzee GTTTTTTCCTTCTTTTTGGCTAATACATAAATCTTGCTTTTGGCAGCCTTGCTATTTTTT 1296

Human Alu (+) **EXON 11**
TTTTTTTTTTTTTTTTTAA-GCCTGTCTGTTTTGTTTTTCTTTACCTTCCCAG CTTCCAGT 1678
Human Alu (–)
TTTTTTTTTTTTTTTTTAA-GCCTGTCTGTTTTGTTTTTCTTTACCTTCCCAG CTTCCAGT 1369
Chimpanzee
TTTTTTTTTTTTTTTTTAAAGCCTGTCTGTTTTGTTTTTCTTTACCTTCCCAG CTTCCAGT 1356

Human Alu (+)
TTCCCAGCCAGTACCAACTATCCAAGGCGAACCTCAGATCCCAGTTGCGACACAACCCTC 1738
Human Alu (–)
TTCCCAGCCAGTACCAACTATCCAAGGCGAACCTCAGATCCCAGTTGCGACACAACCCTC 1429
Chimpanzee
TTCCCAGCCAGTACCAACTATCCAAGGCGAACCTCAGATCCCAGTTGCGACACAACCCTC 1416

Human Alu (+)
GGTTGTTCCAGTCCACTCTGGTGCTCATTTCCTTCCAGTGGGACAGCCGCTCCCTACTCC 1798
Human Alu (–)
GGTTGTTCCAGTCCACTCTGGTGCTCATTTCCTTCCAGTGGGACAGCCGCTCCCTACTCC 1489
Chimpanzee
GGTTGTTCCAGTCCACTCTGGTGCTCATTTCCTTCCAGTGGGACAGCCGCTCCCTACTCC 1476

Human Alu (+)
CTTGCTCCCTCAGTACCCTGTCTCTCAGATTCCCATATCAACTCCTCATGTGTCTACGGC 1858
Human Alu (–)
CTTGCTCCCTCAGTACCCTGTCTCTCAGATTCCCATATCAACTCCTCATGTGTCTACGGC 1549
Chimpanzee
CTTGCTCCCTCAGTACCCTGTCTCTCAAATTCCCATATCAACTCCTCATGTGTCTACGGC 1536

Human Alu (+)
TCAGACAGGTTTCTCATCCCTTCCCATCACAATGGCAGCTGGCATTACTCAGCCTCTGCT 1918
Human Alu (–)
TCAGACAGGTTTCTCATCCCTTCCCATCACAATGGCAGCTGGCATTACTCAGCCTCTGCT 1609
Chimpanzee
TCAGACAGGTTTCTCATCCCTTCCCATCACAATGGCAGCTGGCATTACTCAGCCTCTGCT 1596

Human Alu (+)
CACGTTGGCTTCATCTGCTACAACAGCTGCGATCCCGGGGGTATCAACTGTGGTTCCTAG 1978
Human Alu (–)
CACGTTGGCTTCATCTGCTACAACAGCTGCGATCCCGGGGGTATCAACTGTGGTTCCTAG 1669
Chimpanzee
CACGTTGGCTTCATCTGCTACAACAGCTGCGATCCCGGGGGTATCAACTGTGGTTCCTAG 1656

Human Alu (+)
TCAGCTTCCAACCCTTCTGCAGCCTGTGACTCAGCTGCCAAGTCAGGTTCACCCACAGCT 2038
Human Alu (–)
TCAGCTTCCAACCCTTCTGCAGCCTGTGACTCAGCTGCCAAGTCAGGTTCACCCACAGCT 1729
Chimpanzee
TCAGCTTCCAACCCTTCTGCAGCCTGTGACTCAGCTGCCAAGTCAGGTTCACCCACAGCT 1716

Human Alu (+)
CCTACAACCAGCAGTTCAGTCCATGGGAATACCAGCTAACCTTGGACAAGCTGCTGAGGT 2098
Human Alu (–)
CCTACAACCAGCAGTTCAGTCCATGGGAATACCAGCTAACCTTGGACAAGCTGCTGAGGT 1789
Chimpanzee
CCTACAACCAGCAGTTCAGTCCATGGGAATACCAGCTAACCTTGGACAAGCTGCTGAGGT 1776

Human Alu (+)
TCCACTTTCCTCTGGAGATGTTCTGTACCAG 2129
Human Alu (–)
TCCACTTTCCTCTGGAGATGTTCTGTACCAG 1820
Chimpanzee
TCCACTTTCCTCTGGAGATGTTCTGTACCAG 1807

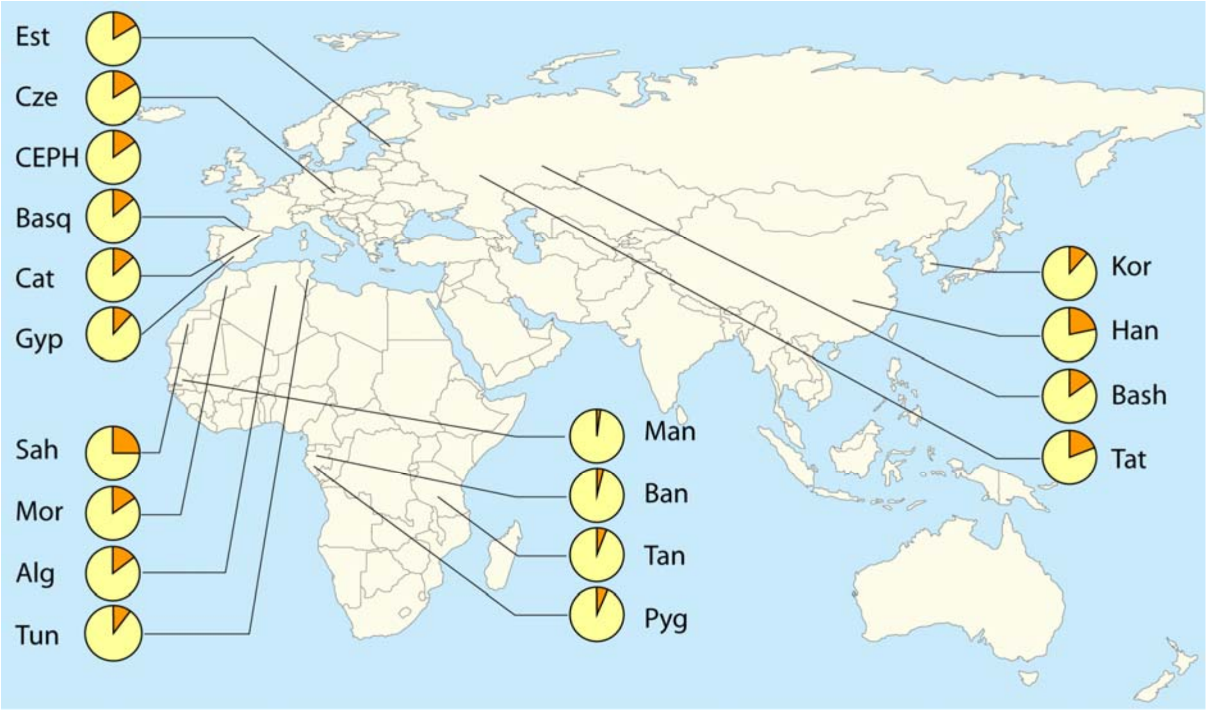

Supp Figure S3. Global map of the allele frequencies of the *WNK1 AluYb8* insertion in studied human population samples from Eurasia and Africa. Pie charts represent the allele frequency of *AluYb8* insertion (orange sector). Lines drawn from the pies point to the geographic affiliation of the respective study population.

Est, Estonians; Cze, Czech; CEPH, CEPH (Utah) families with ancestry from Northern and Western Europe; Basq, Basque; Cat, Catalans; Gyp, Spanish Gypsies; Sah, Saharawi; Mor, Moroccans; Alg, Algerians; Tun, Tunisians; Man, Mandenkalu; Ban, Gabon Bantus; Tan, Tanzanians; Pyg, Gabon Pygmies; Kor, Koreans; Han, Chinese Han; Bash, Bashkir; Tat, Tatars.

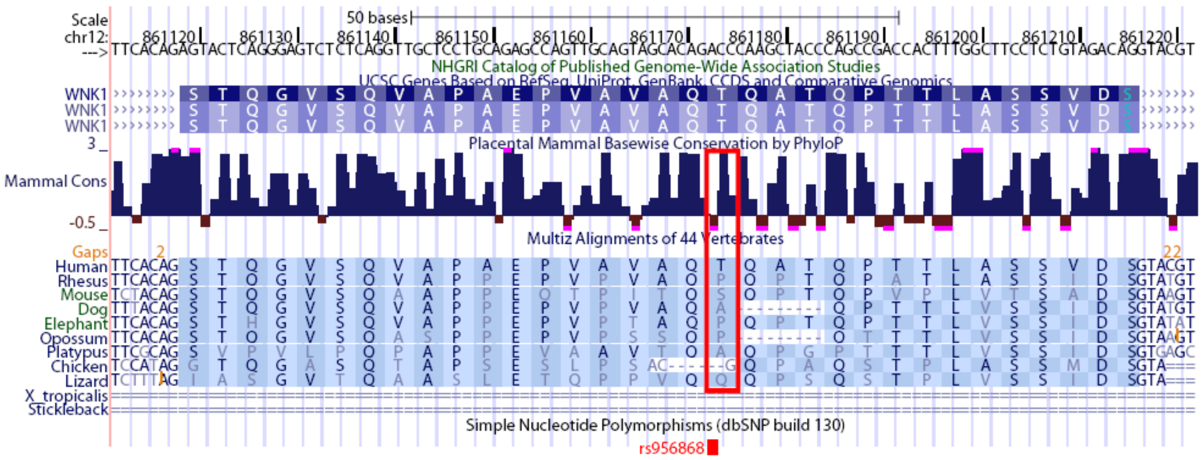

Supp. Figure S4. Amino acid alignment of the *WNK1* exon 13 among vertebrates. Snapshot from UCSC Genome Browser (http://genome.ucsc.edu/; version NCBI36/hg18, Human March 2006) showing the human peptide sequence coded by the *WNK1* exon 13 in comparison to the orthologous region in other species. Red rectangle highlights the human variable position rs956868 coding for two alternative amino acids Proline or Threonine, and exhibiting low evolutionary conservation among vertebrates. The reference sequence of the hg18 (March 2006) carries the minor allele coding for the Threonine (T).

P – proline, S – Serine, A – Alanine, Q – Glutamine, G – Glycine

Supp. Table S1. Characteristics of the *WNK1* (12p13.33; Ensembl ID: ENSG00000060237) and *WNK4* (17q21.31; Ensembl ID: ENSG00000126562) non-coding regions selected for polymorphism screening

No.	*WNK4*/*WNK1* CNS regions	Start[a] position	End[a] position	Sequence type	CNR length (bp)	Rn%[b]	Mm%[c]	DHPLC detected variations; rs	DGGE detected variations; rs	Variation position (dbSNP build130)	Reason for exclusion
WNK4											
**1**	33_WNK4_44	38188209	38188301	non-coding	93	70.3	69.8	ND	none	-	two different Tm melting points for DHPLC
**2**	33_WNK4_62	38193583	38193673	non-coding	91	68	70.3	none	none	-	
**3**	33_WNK4_92	38202314	38202452	UTR	139	72.6	72.7	none	none	-	
**4**	33_WNK4_97	38202700	38202870	non-coding	171	80.7	75.4	A/c; novel	none	38202858	
**5**	33_WNK4_32	38186084	38186188	non-coding	105	77.5	74.8	ND	ND	-	inappropriate primer Tm
**6**	33_WNK4_89	38202123	38202229	non-coding	107	82.4	80.6	ND	ND	-	inappropriate primer Tm
**7**	33_WNK4_91	38202233	38202312	UTR	80	80.5	79.5	none	ND	-	two different Tm melting points for DGGE
**8**	33_WNK4_100	38202944	38203178	non-coding	235	79.8	85.8	none	ND	-	two different Tm melting points for DGGE
**9**	33_WNK4_120	38210504	38210693	non-coding	190	82.8	74.9	none	ND	-	two different Tm melting points for DGGE
**10**	33_WNK4_122	38210713	38210829	non-coding	117	74.5	74.9	none	ND	-	two different Tm melting points for DGGE
**11**	33_WNK4_127	38211778	38211946	non-coding	169	75.6	74.3	none	ND	-	inappropriate primer Tm for DGGE
WNK1											
**12**	34_WNK1_90	831113	831231	non-coding	119	85.8	93.4	none	none	-	
**13**	34_WNK1_139	846970	847070	non-coding	101	87.6	71.6	none	none	-	
**14**	34_WNK1_155	858084	858176	non-coding	93	69.9	80.6	*AluYb8*; novel	*AluYb8;* novel	858212	
**15**	34_WNK1_167	860427	860561	non-coding	135	79.5	79.4	ND	none	-	two different Tm melting points for DHPLC
**16**	34_WNK1_168	860562	860692	non-coding	131	79.5	79.4	none	none	-	
**17**	34_WNK1_208	877345	877471	non-coding	127	75	73.3	none	none	-	
**18**	34_WNK1_222	883173	883364	non-coding	192	72.7	76.1	A/g; rs10849582	none	883437	
**19**	34_WNK1_227	883843	883984	non-coding	142	78.5	76.6	none	none	-	
**20**	34_WNK1_250	889267	889379	UTR	113	77	75.7	A/g; novel	none	889252	
**21**	34_WNK1_12	731433	731594	non-coding	162	77.3	79	ND	ND	-	two different Tm melting points for DHPLC and DGGE
**22**	34_WNK1_37	756329	756526	non-coding	198	75.1	75.5	ND	none	-	genome test negative for DHPLC
**23**	34_WNK1_51	789691	789890	non-coding	200	74.1	75.4	ND	ND	-	genome test negative for DHPLC

No.	*WNK4/WNK1* CNS regions	Start[a] position	End[a] position	Sequence type	CNR length (bp)	Rn%[b]	Mm%[c]	DHPLC detected variations; rs	DGGE detected variations; rs	Variation position (dbSNP build130)	Reason for exclusion
**24**	34_WNK1_53	790344	790469	non-coding	126	92.6	96.2	G/a; rs36092349	ND	790218	two different Tm melting points for DGGE
**25**	34_WNK1_63	808723	808848	non-coding	126	72.6	0	C/t; rs72648627	ND	808848	two different Tm melting points for DGGE
**26**	34_WNK1_67	809338	809429	non-coding	92	0	70.8	ND	ND	-	two different Tm melting points for DHPLC and DGGE
**27**	34_WNK1_81	826028	826198	non-coding	171	82.8	82.8	ND	none	-	genome test negative for DHPLC
**28**	34_WNK1_97	832069	832178	non-coding	110	77	73.7	ND	C/g; rs36052085	832130	genome test negative for DHPLC
**29**	34_WNK1_117	841698	841796	non-coding	99	73.7	75.8	G/a; rs12825084	ND	841868	two different Tm melting points for DGGE
**30**	34_WNK1_120	842050	842208	non-coding	159	72.6	75	ND	none	-	genome test negative for DHPLC
**31**	34_WNK1_146	848285	848553	non-coding	269	87.6	89	ND	ND	-	genome test negative for DHPLC
**32**	34_WNK1_151	850776	850900	non-coding	125	66.9	69	ND	none	-	genome test negative for DHPLC
**33**	34_WNK1_161	858897	858999	non-coding	103	75	70.2	ND	ND	-	two different Tm melting points for DHPLC and DGGE
**34**	34_WNK1_165	860037	860147	non-coding	111	73.9	73	none	ND	-	two different Tm melting points for DGGE
**35**	34_WNK1_174	861501	861617	non-coding	117	67.9	73.1	ND	ND	-	two different Tm melting points for DHPLC and DGGE
**36**	34_WNK1_214	880862	881152	non-coding	291	86.8	85.9	none	ND	-	two different Tm melting points for DGGE
**37**	34_WNK1_218	882302	882503	non-coding	202	75.2	74.1	none	ND	-	two different Tm melting points for DGGE
**38**	34_WNK1_224	883491	883608	non-coding	118	74.6	76.6	ND	none	-	genome test negative for DHPLC
**39**	34_WNK1_246	888231	888518	UTR	288	80.5	80.1	none	ND	-	inappropriate primer Tm
**40**	34_WNK1_259	890715	890782	UTR	68	89.4	85.3	ND	ND	-	two different Tm melting points for DHPLC and DGGE

[a] Start and end positions of the non-coding regions according to Human Genome March 2006 (NCBI Build 36/hg18) assembly.
[b] Sequence identity with *Rattus norvegicus.*
[c] Sequence identity with *Mus musculus.*
CNR - conserved non-coding region; ND - not done

Supp. Table S2. Polymorphism screening primers for DHPLC

No.	*WNK4*/*WNK1* CNS regions	DHPLC forward primer 5´-3´	DHPLC reverse primer 5´-3´	PCR product (bp)	DHPLC Tm
WNK4					
**2**	33_WNK4_62	TGACCTATCTCCCTCCTTGTGAA	ATGACTGTCAGAGCAGACACTGG	323	60°
**3**	33_WNK4_92	CACCTTGGAGGTTTCTTCATCAC	GCTGCTAATGTGGTGTGAGCATA	395	60°
**4**	33_WNK4_97	CACCACATTAGCAGCAACCAA	TCTGTCCTCTACCCACAAGAACA	398	60°
**7**	33_WNK4_91	GATGTTGGCAGGATGGTGAG	ATGAAGATGGTAGGCAGCAGAAC	354	61°
**8**	33_WNK4_100	CTTGTCTTTCACCCTTCATCCAC	CTGTGCAGTGTTTGAGCCTACAG	383	60°
**9**	33_WNK4_120	CCTAGGTTAGGTGATACAACTCATGG	GCAGCATCTGGCCATAGAATAA	385	58°
**10**	33_WNK4_122	CCTGAATTGCCAGCATACCTATC	TCTCTTAGGATCCTCCCATCTCC	378	56°
**11**	33_WNK4_127	TTGCTTGAACCTGGAAGGTGTA	GGTAGAGGTAGAGAAACCTCCATCTG	372	60°
WNK1					
**12**	34_WNK1_90	GTCTGACTTTGATGTCTGCCTTC	CCTCCTCACACAGAATACACCAT	381	55°
**13**	34_WNK1_139	GGCCTCTAACATATCACAGGGTAA	ATGCACAACTGGGTAGTTCTGTG	429	54°
**14**	34_WNK1_155	GGGTAACCAACCCTTGAAGTAGG	GGGTACTTCTCAAGTGATTAGGAGGA	353	56°
**16**	34_WNK1_168	CCAGGAGGGAGTTTAGCACAAG	AATGATTGCTGAGAGCACAGGAG	399	58°
**17**	34_WNK1_208	TGTCTCCAGCTGTATGTGACCTG	CACCGTGCCTAACCAATGACT	486	54°
**18**	34_WNK1_222	GGCCCTACATCCTTAGAAGTCACA	GATCCTGTCCCACTTCCTGTTTC	413	57°
**19**	34_WNK1_227	TGTCTCACCTCCTGCTTTCTCTT	TGTCTGTCACAAGCTAGGCTCAC	368	57°
**20**	34_WNK1_250	CATGTGGAAGGAAATCGTAGGTC	ACGTCTGTTCAAGCCTAGCTCAA	339	58°
**24**	34_WNK1_53	AAGGGACGGAAGATGTGCAA	CTCTTCACCATTATGGAGCTGGA	398	59°
**25**	34_WNK1_63	GCCTGTTTCTTGGAAATGCCTA	CAGCTACACTCTTGGCATTAACACTC	380	57°
**29**	34_WNK1_117	CATGAACAGGCACATTCTACAGG	GCTTCATGTAGGCAGTTCAGCTA	296	59°
**34**	34_WNK1_165	GTCCTGTCTCTTCTGTTGGAACC	TGTGGGAAAGTACCCAGGTGTAG	348	60°
**36**	34_WNK1_214	CTTGATTGGTAAGGAGGGCAAAC	GCCCAAACTTCTGAGGGTAACA	487	59°
**37**	34_WNK1_218	TCTGATGAGTCAGTGCCTACCTG	GGAGCCAATCCATTTCTAATGC	373	58°
**39**	34_WNK1_246	GGAACTGCCTCCTTGCAGAAT	AGAGCTCCACCCACTCGAAATTA	452	60°

Supp. Table S3. Polymorphism screening primers for DGGE

No.	*WNK4*/*WNK1* CNS regions	DGGE forward primer 5´-3´	DGGE reverse primer 5´-3´	PCR product (bp)
WNK4				
**1**	33_WNK4_44	CGCCCGCCGCGCCCCGCGCCCGGCCCGCCGCCCCCGCCC GCGCCCCCGGCGGCAAGTAATCCCATTAAC CCCA	ATAGAAGCGGACGATGTTGGGGT	278
**2**	33_WNK4_62	CGCCCGCCGCGCCCCGCGCCCGGCCCGCCGCCCCCGCCC GTTTTTTTTTTGACCTATCTCCCTCCTTGTGAAA	GCCGACCCCAACTTGTACCAGAACAT	322
**3**	33_WNK4_92	GGGCCCCGGGCCACCTTGGAGGTTTCTTCATCAC	CGCCCGCCGCGCCCCGCGCCCGGCCCGCCGCCCCCGCCC GTTTTTTTTTGATTGCTTAGTGGTTGACTCTTGGC	374
**4**	33_WNK4_97	AACGCCGTGAATCCATATGCCAT	CGCCCGCCGCGCCCCGCGCCCGGCCCGCCGCCCCCGCCC GTTTTGAACATACTGTA ACTCTACTGC	384
WNK1				
**12**	34_WNK1_90	TTCCTTATGCTGTGGGCTGATGTA	CGCCCGCCGCGCCCCGCGCCCGGCCCGCCGCCCCCGCCC GGCATTCTCAAGTTAACAA	341
**13**	34_WNK1_139	CGCCCGCCGCGCCCCGCGCCCGGCCCGCCGCCCCCGCCC GTTTTTTTTTTTTTTTTAGGGAAAGTTAGTTCAATAGTT	CCGAATGAAATCAATCTGTGGGCTCT	329
**14**	34_WNK1_155	ATTGCAGAAATGAAGATC	CGCCCGCCGCGCCCCGCGCCCGGCCCGCCGCCCCCGCCC GTTTTTTTTTTTTTTACAGG GTACTTCTCAAGTGATTAGG	379
**15**	34_WNK1_167	CGCCCGCCGCGCCCCGCGCCCGGCCCGCCGCCCCCGCCC GTTTTTTTTTTTTTTTTTTT CACTACATCCTCCCAGCAAG	AGGACCCCACATTGGCATTTCATTC	263
**16**	34_WNK1_168	CGCCCGCCGCGCCCCGCGCCCGGCCCGCCGCCCCCGCCC GCGTATATATGTAATTGGACAAC	AGCCAGCATCTTACCAAGAGTGT	433
**17**	34_WNK1_208	CGCCCGCCGCGCCCCGCGCCCGGCCCGCCGCCCCCGCCC GTTTTTTTTTTTTAGACTTAGTGAATACAATGGC	GGCCGGTTGCAATTATGACTAAGTCA	248
**18**	34_WNK1_222	CGGGCGTGGTATTACCCCTGTTGTTCAA	CGCCCGCCGCGCCCCGCGCCCGGCCCGCCGCCCCCGCCC GGGCTATTATATTTCCACATCCC	415
**19**	34_WNK1_227	TGTCTCACCTCCTGCTTTCTCTT	CGCCCGCCGCGCCCCGCGCCCGGCCCGCCGCCCCCGCCC GTTTTTTTTTTGAATAGGATACAGAGATAGATGG	328
**20**	34_WNK1_250	CGCCCGCCGCGCCCCGCGCCCGGCCCGCCGCCCCCGCCC GTTTTTTTTTTTCAGTGGTTATGCCAAAGGGAAAT	GGGCCCACCATTCTTTTAAGTTGTCCAA	245
**22**	34_WNK1_37	GTGAACTATAGAGAATTGACC	CGCCCGCCGCGCCCCGCGCCCGGCCCGCCGCCCCCGCCC GTTTTTTTTTTTTTTTTTTTTGCACAGATGCTGACTAATTTT	324

**No.**	***WNK4*/*WNK1* CNS regions**	**DGGE forward primer 5´-3´**	**DGGE reverse primer 5´-3´**	**PCR product (bp)**
**27**	34_WNK1_81	GGTTTCCAATGGGATCATAGGGT	CGCCCGCCGCGCCCCGCGCCCGGCCCGCCGCCCCCGCCC GTTTTTTTTTTTTTTTCAGTAAAAAGGTAGGGTGAATT	334
**28**	34_WNK1_97	AGTGGAATGAGAGGGCTGTAT	CGCCCGCCGCGCCCCGCGCCCGGCCCGCCGCCCCCGCCC GGGGACATGTAATAGAAAGCTG	340
**30**	34_WNK1_120	CGCCCGCCGCGCCCCGCGCCCGGCCCGCCGCCCCCGCCC GTTTTTTTTTTTTTTTTTGGCGAGAAGAGTTTTTCAAAA	TCCAACACAGACTTTAAAAGGCA	283
**32**	34_WNK1_151	TCATCTTCCCAACCCATACAA	CGCCCGCCGCGCCCCGCGCCCGGCCCGCCGCCCCCGCCC GTTTTTTTTTTGAGATTTTTCTGAAACACAG	264
**38**	34_WNK1_224	CCGGCCCTACTGTGGTGGGGTTGCTAATGCAT	CGCCCGCCGCGCCCCGCGCCCGGCCCGCCGCCCCCGCCC GCAGATATAGTTCTACAGAATG	342

Supp. Table S4. Sequences of oligonucleotide primers and probes used in PCR and RT-PCR reactions

Primer ID	Sequence 5´- 3´	5´modification	3´modification
**A**. Amplification of genomic area covering exons 10 and 11			
WNK1_Seq1_F	TGGGGTGAGGGAGATAATTGGGTG		
WNK1_Seq_R	ACTCTGTGGTGCCCTCTTTTGCT		
**B**. Sequencing primers			
WNK1_Seq3_F	TCAGGTAGTGGAGAGGGATGATTG		
WNK1_Seq4_F	ACCCCTTGTCTATGGAAGGGTCC		
WNK1_Seq5_F	CCTTCCAGTGGGAGAGCCGC		
WNK1_Alu_F	CGGCCTCCCAAAGTGCTGGG		
**C**. RT-PCR primers and probes			
WNK1_Exon10_F	CCACAAGTCTTGCCTCAAGTATCA		
WNK1_Exon12_R	GGAAGAGGGAGCAATATTTGAATC		
WNK1_Exon13_R	TGCAGGAGCAACCTGAGAGA		
Ex–11+12_probe	TGGAAAACAGGGCTTC	[a]6-FAM™	[b]MGBNFQ
Ex–11–12_probe	CTGGAAAACAGAGTACTC	6-FAM™	MGBNFQ

[a] Phosphoramidite
[b] Minor groove binding non-fluorescent quencher

Supp. Table S5. Characteristics of the individuals used for *WNK1* expression profiling in leucocytes

Genotype	Gender	Age	SBP	DBP	BMI
–/–	female	40	100	60	23.0
	female	36	105	70	21.3
	female	46	118	83	23.0
Alu/–	female	51	118	75	27.0
	female	43	113	73	25.6
	female	47	127	82	20.8
Alu/Alu	female	35	156	100	21.8
	female	64	190	110	31.6
	female	57	165	100	26.3

SBP – Systolic Blood Pressure; DBP – Diastolic Blood Pressure;
BMI – Body Mass Index

Supp. Table S6. Polymorphisms detected in screened conserved non-coding regions of human *WNK1* and *WNK4* genes

Analyzed regions[a]			Detected variants				
Gene	Location	Length of fragment (bp)	Position of the variant[b]	Alleles[c]	HYPEST (hom/het)[d]	CADCZ (hom/het)[d]	Validation/ rs-number[b]
WNK1	Intron 1	398bp	Chr.12; 790218	G/a	1/3	0/1	rs36092349
WNK1	Intron 3	380bp	Chr.12; 808848	C/t	0/1	0/0	rs72648627
WNK1	Intron 4	340bp	Chr.12; 832130	C/g	na/6	na/6	rs36052085
WNK1	Intron 8	296bp	Chr.12; 841868	G/a	0/0	0/2	rs12825084
WNK1	Intron 10	353bp	Chr.12; 858212	D/i	1/7	3/9	novel
WNK1	Intron 26	413bp	Chr.12; 883437	A/g	0/0	0/4	rs10849582
WNK1	3´UTR	339bp	Chr.12; 889252	A/g	0/1	0/0	novel
WNK4	5´UTR	398bp	Chr.17; 38202858	A/c	0/0	0/1	novel

[a] Details of the analyzed regions and conditions of the DHPLC/DGGE assays are given in Supp. Table S1.
[b] Positions of the variants and rs-numbers of the validated SNPs according to the dbSNP build 130; Human Genome March 2006.
[c] Major (capital letter) and minor (lower case letter) alleles according to the coding strand.
[d] Number of homozygotes/ heterozygotes (hom/het) of each identified polymorphism among the screened HYPEST (n=22) and CADCZ (n=24) patients.
D/i – deletion/insertion variant of *WNK1 AluYb8*
na – not applicable as DGGE method was incapable to distinguish CC and GG homozygotes

Supp. Table S7. Allele frequencies of the *WNK1 AluYb8* insertion in general human population samples

		Number of individuals				*AluYb8* allele
Region	Population	Alu/Alu[a]	Alu/–[b]	–/–[c]	total	frequency
Europe	Estonians	4	25	71	100	16.5%
	Czechs	1	14	35	50	16.0%
	CEPH (Utah) families	2	5	23	30	15.0%
	Basques	0	14	36	50	14.0%
	Catalans	0	11	30	41	13.4%
	Spain Gypsies	0	12	38	50	12.0%
Asia	Chinese Han	2	7	16	25	22.0%
	Koreans	1	8	34	43	11.6%
	Tatars	1	16	30	47	19.1%
	Bashkirs	2	10	35	47	14.9%
North-Africa	Moroccans[d]	2	22	60	84	15.5%
	Saharawi	6	13	31	50	25.0%
	Tunisians	1	8	39	48	10.4%
	Algerians	0	14	34	48	14.6%
Sub-Saharan Africa	Mandenkalu	0	1	23	24	2.1%
	Tanzanians	0	2	15	17	5.9%
	Bantus	0	4	46	50	4.0%
	Pygmies	0	7	43	50	7.0%

[a] Homozygotes and [b] Heterozygotes for the *WNK1 AluYb8* insertion.
[c]Wild-type genotype without the *WNK1 AluYb8* insertion.
[d] Samples were collected from five different cities in Morocco (Oujda, Casablanca, Rabat, Chefchaouen, Nador).
Mandenkalu and Chinese Han samples were purchased from the HGDP-CEPH Human Genome Diversity Cell Line Panel (http://www.cephb.fr/HGDP-CEPH-Panel/). Korean samples were kindly shared by Dr. Woo Chul Moon (Good-Gene Inc. Seoul, Korea).

Supp. Table S8. Association testing of the *WNK1* intron 10 *AluYb8* with hypertension

	Sample size: total and stratified by genotype (Alu/Alu; Alu/–; –/–)		*AluYb8* allele frequency (%)		Association testing[a]	
Sample	Cases	Controls	Cases	Controls	OR (CI 95%)	*P*-value
HYPEST[b]: essential hypertension/ normotensive controls						
all	673 (21/197/455)	601 (13/148/440)	17.70	14.51	1.24 (0.91, 1.69)	$1.66x10^{-1}$
women	445 (16/140/289)	439 (11/109/319)	19.28	14.97	1.17 (0.81, 1.69)	$4.03x10^{-1}$
men	228 (5/57/166)	162 (2/39/121)	14.63	13.27	1.42 (0.79, 2.52)	$2.38x10^{-1}$
CADCZ[b]: CAD patients with hypertension/ normotensive controls						
all	266 (10/71/185)	480 (9/129/342)	17.11	15.31	1.23 (0.89, 1.71)	$2.14x10^{-1}$
women	86 (5/21/60)	251 (5/61/185)	18.02	14.14	1.37 (0.83, 2.29)	$2.22x10^{-1}$
men	180 (5/50/125)	229 (4/68/157)	16.67	16.60	1.14 (0.74, 1.75)	$5.57x10^{-1}$
BRIGHT[c]: extreme family based hypertension/ normotensive controls						
all	2242 (54/544/1644)	1639 (43/391/1205)	14.54	14.55	1.00 (0.88, 1.14)	$9.95x10^{-1}$
women	1320 (40/319/961)	1002 (29/249/724)	15.11	15.32	0.99 (0.84, 1.16)	$8.85x10^{-1}$
men	922 (14/225/683)	637 (14/142/481)	13.72	13.34	1.02 (0.83, 1.26)	$8.66x10^{-1}$

[a] Logistic regression (additive model, age and gender as covariates) were used to test association with HYP.

[b] Cases: subjects under antihypertensive treatment or untreated subjects SBP ≥160 mmHg and/or DBP ≥100 mmHg; Controls: subjects with SBP ≤140 mmHg and DBP≤90 mmHg, receiving no antihypertensive medication.

[c] Cases: patients originating from severely hypertensive families, under antihypertensive treatment and with BP readings ≥150/100 mmHg based on one reading or ≥145/95 mmHg based on the mean of three readings; Controls: normotensive subjects with BP <140/90 mmHg, receiving no antihypertensive medication.

Alu/–, Alu/Alu *WNK1 AluYb8* hetero- and homozygote carriers; –/– wild-type homozygotes; CAD, coronary artery disease; OR, odds ratio; CI, confidence interval

Supp. Table S9. Linkage disequilibrium (r^2) between the *WNK1 AluYb8* and common SNPs in the *WNK1* gene previously reported in the BRIGHT study (data from Newhouse et al., 2009)

SNP	Location	Position[a]	MAF[b]	r^2
rs7137188	5′	724592	0.48	0.0003
rs1468326	5′	727762	0.11	0.0029
rs3088353	5′	732901	0.46	0.1908
rs11064519	Intron 1	741472	0.34	0.0044
rs2369402	Intron 1	748925	0.21	0.0476
rs2107612	Intron 1	758581	0.27	0.0723
rs2107613	Intron 1	758689	0.23	0.0543
rs11608756	Intron 1	760094	0.39	0.2561
rs11064524	Intron 1	760163	0.25	0.0423
rs11064527	Intron 1	761286	0.16	**0.8216***
rs11064536	Intron 1	775843	0.17	0.0345
rs765250	Intron 1	778544	0.31	0.0800
rs12314329	Intron 1	779992	0.08	0.0176
rs11611246	Intron 4	809741	0.22	0.0294
rs6489756	Intron 4	834767	0.47	0.1800
rs12816718	Intron 6	840061	0.15	**0.9213***
rs2286007	Exon 8	841552	0.07	0.0140
rs11611231	Intron 8	844587	0.10	0.0095
rs4980973	Intron 9	853307	0.13	0.0248
rs4980974	Intron 9	855289	0.44	0.1277
AluYb8	Intron 10	858213	0.15	1
rs880054	Intron 10	858819	0.41	0.1286
rs956868	Exon 13	861173	0.14	**0.9753***
rs953361	Intron 22	872068	0.40	0.0878
rs2301880	Intron 23	874098	0.24	0.0602
rs2286026	Intron 26	885434	0.41	0.1247
rs2286028	Intron 26	885730	0.20	0.0371
rs2277869	Intron 26	887171	0.16	0.0233
rs11571461	3′	896580	0.07	0.0150

[a] Positions of the SNPs according to the dbSNP build 130; Human Genome March 2006.
[b] MAF, minor allele frequency
*** r^2>0.8**

Table reference:

Newhouse S, Farrall M, Wallace C, Hoti M, Burke B, Howard P, Onipinla A, Lee K, Shaw-Hawkins S, Dobson R, Brown M, Samani NJ, Dominiczak AF, Connell JM, Lathrop GM, Kooner J, Chambers J, Elliott P, Clarke R, Collins R, Laan M, Org E, Juhanson P, Veldre G,

Viigimaa M, Eyheramendy S, Cappuccio FP, Ji C, Iacone R, Strazzullo P, Kumari M, Marmot M, Brunner E, Caulfield M, Munroe PB. 2009. Polymorphisms in the WNK1 gene are associated with blood pressure variation and urinary potassium excretion. *PLoS One 4*: e5003.

Supp. Table S10. Association of the *WNK1 AluYb8* and SNPs rs11064527, rs12816718, rs956868 with blood pressure in the BRIGHT

Trait		rs11064527 (Intron 1)		rs12816718 (Intron 6)		*AluYb8* indel (Intron 10)		rs956868 (Exon 13) Thr1316Pro (NP_001171914)	
SBP		Beta (SE)[b]	*P*-value	Beta (SE)	*P*-value	Beta (SE)	*P*-value	Beta (SE)	*P*-value
	all	1.16 (0.49)	$1.67x10^{-2}$	1.15 (0.50)	$2.18x10^{-2}$	1.50 (0.51)	$3.01x10^{-3}$	1.47 (0.52)	$4.90x10^{-3}$
	women	1.26 (0.67)	$5.83x10^{-2}$	1.22 (0.68)	$7.55x10^{-2}$	1.60 (0.68)	$1.91x10^{-2}$	1.48 (0.70)	$3.46x10^{-2}$
	men	1.00 (0.67)	$1.40x10^{-1}$	1.07 (0.70)	$1.27x10^{-1}$	1.27 (0.73)	$8.12x10^{-2}$	1.41 (0.75)	$6.11x10^{-2}$
DBP									
	all	0.61 (0.34)	$7.64x10^{-2}$	0.52 (0.35)	$1.40x10^{-1}$	0.78 (0.36)	$2.99x10^{-2}$	0.72 (0.37)	$5.15x10^{-2}$
	women	1.07 (0.46)	$1.89x10^{-2}$	0.93 (0.47)	$4.98x10^{-2}$	1.22 (0.47)	$9.48x10^{-3}$	1.06 (0.48)	$2.88x10^{-2}$
	men	-0.14 (0.51)	$7.85x10^{-1}$	-0.10 (0.52)	$8.43x10^{-1}$	-0.03 (0.55)	$9.49x10^{-1}$	0.09 (0.56)	$8.70x10^{-1}$

The association testing was performed using the BRIGHT normotensive individuals (n=1421) genotyped in the current study for the *AluYb8* insertion, and in the previous study for rs11064527, rs12816718, rs956868 (Newhouse et al., 2009). Statistical analysis was identical to the conditions described in Materials and Methods for the association testing with quantitative variables, SBP and DBP (linear regression, additive model, age and gender as covariates).
SBP, DBP, systolic and diastolic blood pressure; SE, standard error
